# Neuronal tuning aligns dynamically with object and texture manifolds across the visual hierarchy

**DOI:** 10.1038/s41593-026-02207-1

**Published:** 2026-03-10

**Authors:** Binxu Wang, Carlos R. Ponce

**Affiliations:** 1 Kempner Institute for the Study of Natural and Artificial Intelligence, Harvard University, Allston, MA USA; 2 Department of Neurobiology, Harvard Medical School, Boston, MA USA

**Keywords:** Object vision, Pattern vision

## Abstract

Visual neurons respond to a vast range of images, from textures to objects, but the rules linking these responses remain unclear. Although tuning to simple features is well established in the primary visual cortex, this framework breaks down in higher areas, where neurons encode diverse and unpredictable features. To ask what features neurons prioritize, we used generative models (deep networks that synthesize new images from a learned latent space), allowing neurons in V1, V4 and the posterior inferotemporal cortex (PIT) to guide image synthesis through closed-loop optimization. We compared models that emphasize texture versus those that emphasize object structure. Although V1 and V4 aligned more strongly with texture-based spaces, many PIT neurons responded equally well to both types of optimized images, revealing a focus on shared local motifs rather than whole-object templates, and this alignment to objects emerged later in their response. These findings reveal coding principles across the ventral stream and clarify the limits of current vision models.

## Abstract (web summary)

Visual neurons drive two AI models to compete in generating neuron-preferred features. The models form shared local motifs, while neuronal preference shifts from texture to object structure over time, revealing dynamic changes in visual coding.

## Main

**Fig. 1:**
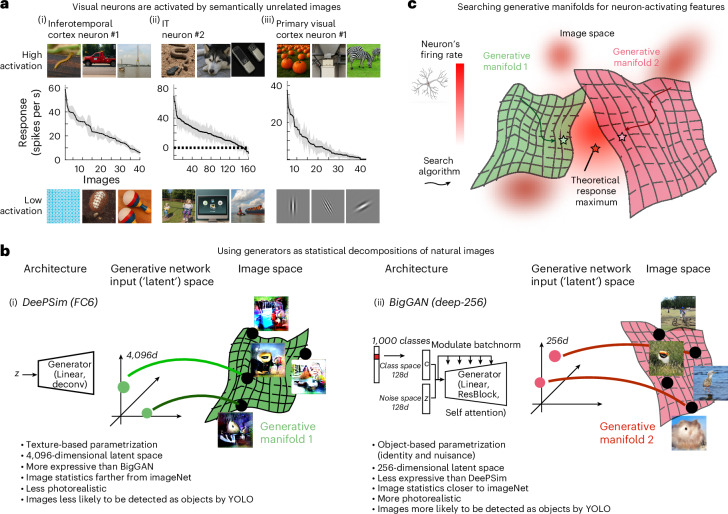
Texture and object image manifolds as parameterized by DeePSim and BigGAN. **a**, Most visual neurons respond strongly to sets of natural images with little semantic relation. Each curve shows responses to randomly sampled images (top, most activating; bottom, least activating). Shaded areas: s.e.m. **b**, Architecture and image statistics of the two generative models. (i) DeePSim uses an up-convolutional architecture that generates texture-like, less photorealistic images with a 4,096-dimensional latent space. (ii) BigGAN combines class and noise embeddings into a 256-dimensional latent space and produces object-centric, photorealistic images. Each generator defines a continuous image manifold mapping latent codes to naturalistic images. **c**, Conceptual framework: neuronal firing rates guide searches across generative manifolds to locate response maxima. Parallel optimizations in DeePSim (texture manifold) and BigGAN (object manifold) reveal complementary aspects of neuronal tuning across the visual cortex.

To understand the visual brain, we must examine what activates its constituent neurons. For decades, highly activating images have been used as hypotheses about the types of visual information and latent factors encoded by individual neurons and cortical columns. Such factors have ranged from physical properties such as orientation^1,2^, direction^3,4^ and depth^5,6^, to high-level categorical examples such as faces^7^, bodies^8^ and places^9^. A common aspiration was that by defining the function of neurons within each hypothesis-driven subspace, we could generalize neuronal tuning functions across natural scenes^10^. However, when neurons are probed with broad image sets, their responses often defy expectations. Most visual cortex neurons respond strongly to images that share little to no semantic relationship. For instance, a single neuron might respond robustly to a picture of a centipede, a truck and a bridge (Fig. 1a(i,ii)). Even primary visual cortex (V1) neurons, typically described as tuned to oriented contours, often show higher activity to specific natural images, suggesting that other triggering features may be present but not obvious to the human eye (Fig. 1a(iii)). Thus, one hypothesis is that visual neurons encode sub-categorical ‘critical’ features^11^ that recur across objects and scenes. Such features are difficult to define a priori, perhaps because humans, when viewing whole objects and crowded scenes, lose the facility to isolate constituent local features^12–14^. The question becomes how we explain the capacity of neurons to extract meaningful signals from globally different images.

One approach to identify these activating ‘critical features’ is to probe neurons with synthetic images from deep generative networks. Generative adversarial networks (GANs) learn statistical regularities in natural scenes and can produce new examples^15^. They can smoothly interpolate between known features across unknown intermediate patterns that might be of interest to the neuron. GANs map vectors from a latent space to images (Fig. 1b), parametrizing image manifolds. Searching within these manifolds (Fig. 1c) allows us to turn neuronal activity into activating image sets containing critical features and to map the broader response range and tuning of individual neurons and microclusters (that is, cortical columns or other forms of multi-unit activity). This approach has been used to study units in deep networks^16^ and neurons in the primate visual cortex^17–19^; more broadly, generative networks have become essential tools in neuroscience research^20–24^. Yet the image priors of such models matter. Our earlier work relied on DeePSim^25^, a powerfully expressive model that approximates photographs and extrapolates beyond its training distribution (for example, color-inverted or scrambled images; see supplementary Figs. S19 and S20 in ref. ^16^). However, most DeePSim synthetic images do not look like photographs, lacking full objects or semantic meaning. By contrast, BigGAN was trained on ImageNet^26^ to generate object-centered, photorealistic images with explicit category conditioning^27^. However, this model does not readily produce object-free images, and it is not certain how well it can produce images with features and statistics outside of its training distribution. Testing neurons with both generators thus offers complementary views of visual feature representation. Here, we used our understanding of the geometry of generative networks’ latent spaces^28^ and their suitable evolutionary algorithms^29^ to optimize images that activate neurons along the macaque occipito-temporal pathway. We compared how DeePSim and BigGAN spaces suited the tuning of neurons in V1, V4 and PIT. These generators span large image manifolds with contrasting priors, providing strong tests of neuronal adaptability. We assessed alignment between neuronal tuning and each generative space based on three factors: optimization ease (reflecting smoother tuning landscapes), activation before and after image optimization (proximity to tuning peaks) and optimization speed (faster convergence indicating better alignment). This alignment reveals principles underlying ventral stream coding.

## Results

Our goal was to compare how visual neurons responded when tested with two different types of generative models. These models represent contrasting priors or ‘visual languages’: DeePSim produces textural patterns without coherent objects, whereas BigGAN produces object-like images with photorealistic detail. By optimizing images in both spaces simultaneously, we asked whether neurons across the ventral stream aligned better with texture-based or object-based representations.

We have organized the Results section into five stages. First, we describe the properties of DeePSim and BigGAN, highlighting how their images differ and the hyperparameter tuning specific to each latent space (Stage 1). Next, we show that neurons can guide optimization in both spaces, often driving different-looking images that nonetheless share critical local features (Stage 2). We then compare alignment across the ventral hierarchy, showing that V1 and V4 neurons aligned better with the textural space, whereas PIT neurons aligned well with both (Stage 3). We extend this analysis to neural dynamics, revealing that PIT neurons initially responded more to textures but later favored object-based features (Stage 4). Finally, we examine tuning function shapes, showing that neurons can display bell-shaped or ramp-like tuning depending on how close optimization comes to their preferred features (Stage 5). Together, these stages outline how neurons align with different image spaces, shedding light on the organization of the ventral visual hierarchy and model limitations.

### Characterizing generators and adapting closed-loop optimization

**Extended Data Fig. 1:**
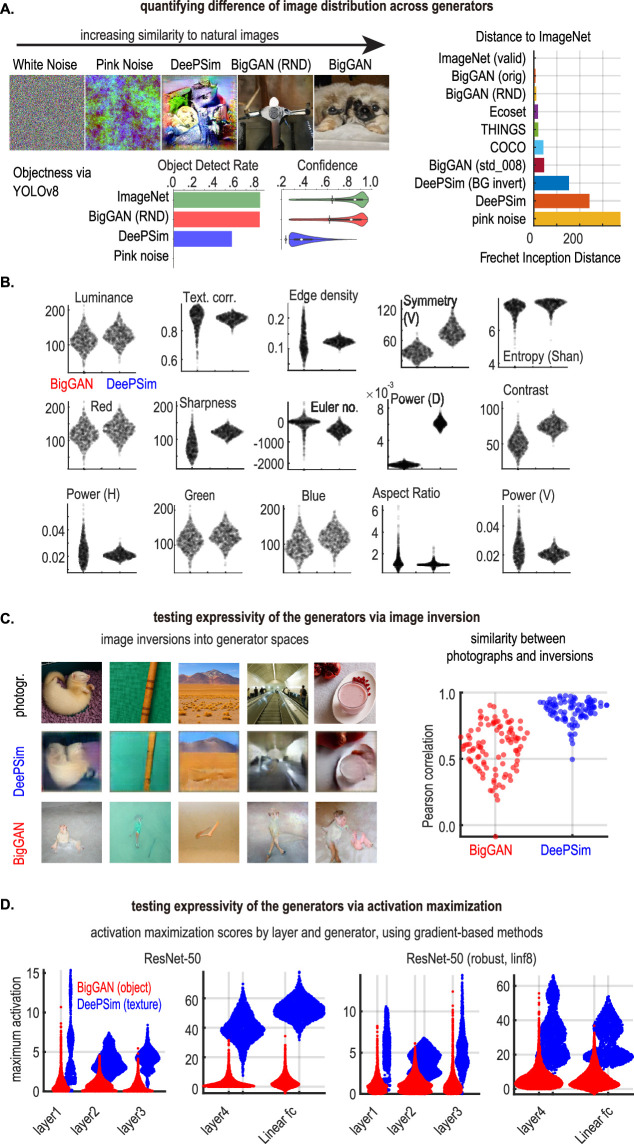
Exploring the image statistics and representational flexibility of the generators. **A**. Left: Representative synthetic images generated from DeePSim and BigGAN, illustrating visual characteristics produced by each generator. Right: Fréchet Inception Distance (FID) comparing each generator’s image distribution to natural image datasets. BigGAN more closely matched natural image statistics than DeePSim. For reference, the FID between ImageNet and other natural image datasets (EcoSet, THINGS-object, MS-COCO) is also shown. Each FID value reflects the empirical distance computed from a fixed sample of generated images (n = 50,000 / condition] synthetic images per generator) and an equal number of natural comparison images. Bottom: Objectness quantification using the YOLOv8 object-detection model. “Object detect rate” shows the fraction of samples in which at least one object was detected, and “detection confidence” reports the maximum confidence score per image. Sample sizes were the same as for the FID analysis. BigGAN and ImageNet showed highly similar objectness distributions, whereas DeePSim exhibited markedly reduced object detectability. Violin plots show the full distribution of values. Box plots inside violins indicate the median (center line), the 25th and 75th percentiles (lower and upper box edges), and whiskers extending to 1.5× the interquartile range or to the most extreme datapoint within that range. Outliers beyond the whiskers are shown as individual points. **B. Low-level statistics of images optimized in DeePSim and BigGAN spaces**, optimizing the same hidden units in AlexNet. Specifically, images were optimized for units in AlexNet conv5 (with receptive fields placed at the center, N = 91 units); a preferred image for each unit was concurrently optimized using the DeePSim- and the BigGAN generators. Analyses are based on all images in the last evolved generation (N = 3640 total). Each point represents a sampled image from the latent space of each generator. Values are mean ± SEM. **C. Image Inversion Experiment**. As the next test of representational flexibility, we replaced activation maximization with a different objective and process: an image inversion test. The goal was to determine how well DeePSim and BigGAN could approximate randomly sampled photographs by optimizing their latent codes. By comparing the similarity between the randomly sampled images and their reconstructions from each GAN, we examined differences in representational flexibility. For this experiment, we sampled 101 photographs randomly from the EcoSet dataset across all categories. Synthetic images were optimized using the Adam algorithm to minimize the mean squared error (MSE) between the original image and the GAN reconstruction over 401 iterations. Per visual examination, many images were well-matched by DeePSim, whereas BigGAN often matched overall color but instead of objects or textured scenes, it frequently placed a single central humanoid object in the image center (Fig. 1). We measured the similarity between original images and their GAN-generated inversions using Pearson correlation (over pixel space). DeePSim achieved a median similarity of 0.89 ± 0.01 (SE), while BigGAN achieved a median similarity of 0.60±0.02. This difference was statistically significant (P < 0.00001, Wilcoxon signed-rank test, N = 101, Fig. 1). Overall, these results suggest that, given a fixed optimization process and reconstruction objective, DeePSim provides substantially more accurate approximations compared to BigGAN, likely reflecting differences in the flexibility of their respective latent spaces. (*right)* The first row shows randomly sampled images from the EcoSet dataset. The second and third rows show gradient-based reconstructions by DeePSim (blue) and BigGAN (red). (*left*) Similarity to original images. Scatterplots show the Pearson correlation between the original randomly sampled images and their GAN-based reconstructions for DeePSim (blue) and BigGAN (red). **D**. Activation maximization scores. To measure how generators compared in their ability to represent different types of images, we performed a series of computational experiments. First, we used gradient-based methods to optimize images for hidden units. This allowed us to isolate the role of optimization type and assess each generator’s inherent capacity for activation maximization. We used two ResNet architectures, ResNet-50 and a ResNet-50 variant trained to withstand adversarial attacks (ResNet-50-robust or ResNet-50linf826), conducting 97,730 experiments using units from multiple layers. In each experiment, we optimized the latent vectors of each generator to maximize the activation of a given individual unit. The analysis involved several optimization strategies (for example, Adam variants, stochastic gradient descent variants), different learning rates (for example, 0.001, 0.01, 0.1), and other settings such as Hessian-based modifications (for example, Adam001Hess, SGD001Hess). We found that DeePSim consistently led to higher activation scores compared to BigGAN, regardless of model (vanilla or robust), layer, or optimization method. Across all experiments, the median activation score achieved through DeePSim was 5.22 ± 0.04, while the median activation score achieved through BigGAN was 1.115 ± 0.005. These values were statistically different per a Wilcoxon rank sum test (P < 0.00001, r = −0.533). Each violin plot shows the maximum activation scores achieved by ResNet-50 (left) and ResNet-50linf8 (robust) hidden units after gradient-based optimization of images, using BigGAN (red) and DeePSim (blue). X-axis labels show the layer of the hidden units tested. Source data

Before turning to the biology, we first tested the visual impression that DeePSim generated abstract textural patterns, whereas BigGAN produced object-like, naturalistic images. Quantitative analyses showed that BigGAN’s image distribution was closer to real object photographs (for example, Fréchet inception distance (FID) to ImageNet of 10 vs 197 for DeePSim; Extended Data Fig. 1a) and different for many low-level metrics (Extended Data Fig. 1b). To test expressivity, we performed an image inversion task to assess how well each generator could reconstruct random photographs. DeePSim reconstructed them much more faithfully than BigGAN, which tended to produce object-like outputs regardless of the target^30^ (Extended Data Fig. 1c). Thus, DeePSim’s latent space was more flexible, whereas BigGAN traded flexibility for object-centric priors, a contrast that is ideal for probing the visual cortex.

Next, we tuned hyperparameters in silico using hidden units in convolutional neural networks (CNNs), which share key properties with visual neurons^31^. Real-time image optimization in vivo relies on a closed loop between an image generator and a search algorithm^32^; here, we used the covariance matrix adaptation evolutionary strategy (CMA-ES)^17,18,33^. To adapt it to BigGAN, we adjusted the exploration step using CNN units^29^ before in vivo testing. With this adjustment (step of 0.06–0.4 vs 3.0 for DeePSim), the same algorithm (CMA-ES) optimized successfully in both generators without further changes (see Methods). Given that this process used evolutionary algorithms, we refer to each optimization run as an evolution.

**Extended Data Fig. 2:**
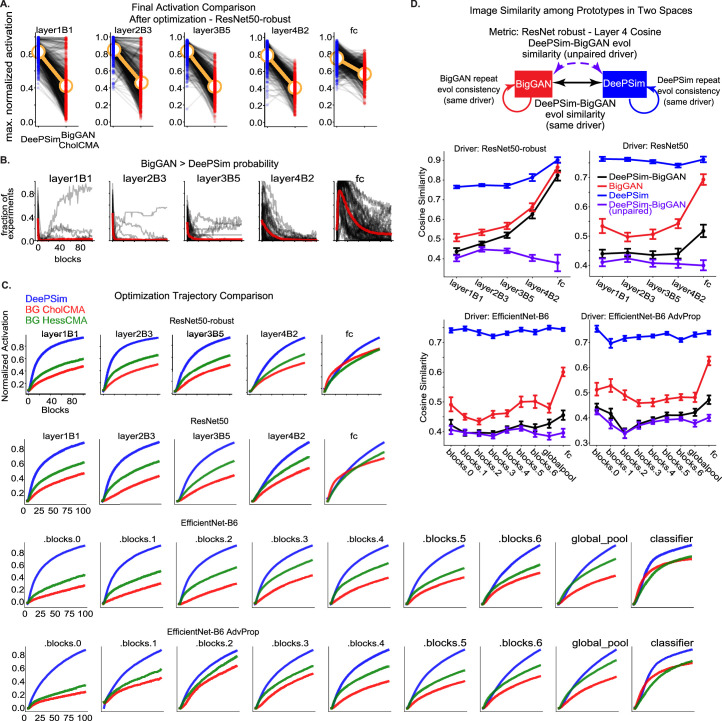
Win Rate and Convergence Time Constant Analysis. **A. Final activation comparison** post optimization in DeePSim and BigGAN space, for units in ResNet50-robust. **B. Win rate of BigGAN for ResNet50-robust units**. The thin black trace represents the averaged win rate for each unit across 10 repetitions. The red curve shows the average win rate curve for each layer, averaged across all units. **C. Evolution trajectory in DeePSim and BigGAN Space for all CNN networks**. Same format as **B**. Top to bottom: ResNet50-robust, ResNet50, EfficientNet-B6, EfficientNet-B6 AdvProp, 50 units were sampled from each major layers, and 10 repeated evolutions were conducted in both DeePSim and BigGAN space. Consistently, driven by the same unit, DeePSim evolution reached higher activation than BigGAN, with the gap closer for deeper units in the network. Shaded area shows SEM across all runs, sometimes too small to be seen. **D. Image similarity of paired DeePSim and BigGAN prototypes from in silico evolutions**. Similarity between prototypes was higher when their synthesis was driven by the same driver than when driven by shuffled pairs. Drivers were units from ResNet50. The error bar shows the 95% confidence interval of the mean, across pairs. N = 6400. Source data

We also conducted parallel evolution experiments on CNN units, selecting driver units with receptive fields at the image center and then running ten evolutions per generative model. Across all units, optimization in the texture manifold consistently produced higher activations than in the object manifold, regardless of optimization algorithm or layer in the network (DeePSim > BigGAN, paired *t*-test, *t*_499_ > 24.7, *P* < 4.7 × 10^−89^ for all layers; Extended Data Fig. 1d for gradient ascent and Extended Data Fig. 2 for evolutionary algorithms). This texture preference persisted even in the final object classification layer, reflecting the texture bias of vision networks^34^. We then asked the question of whether this prediction would hold for the ventral stream.

### Neuron-guided image synthesis in two generative spaces

Having adapted this new generator for in silico optimization, we next compared how visual cortex neurons directed image optimization in each generative space. We conducted parallel evolution experiments using two male macaque monkeys (monkeys A and B; later experiments included two more: monkeys C and D), implanted with chronic floating microelectrode arrays in three visual cortical areas (V1, posterior to the lunate sulcus; V4, on the prelunate gyrus; and PIT, anterior to the inferior occipital sulcus). Each day, after sorting and classifying neuronal signals into single-units or multi-units, we mapped receptive fields and selected a visually responsive unit as the driver unit of the session. Using its firing rate as the optimization target, we conducted two parallel evolutions (threads): one searching within the BigGAN manifold and the other within the DeePSim manifold, each with a separate optimizer configured for its space.

**Fig. 2:**
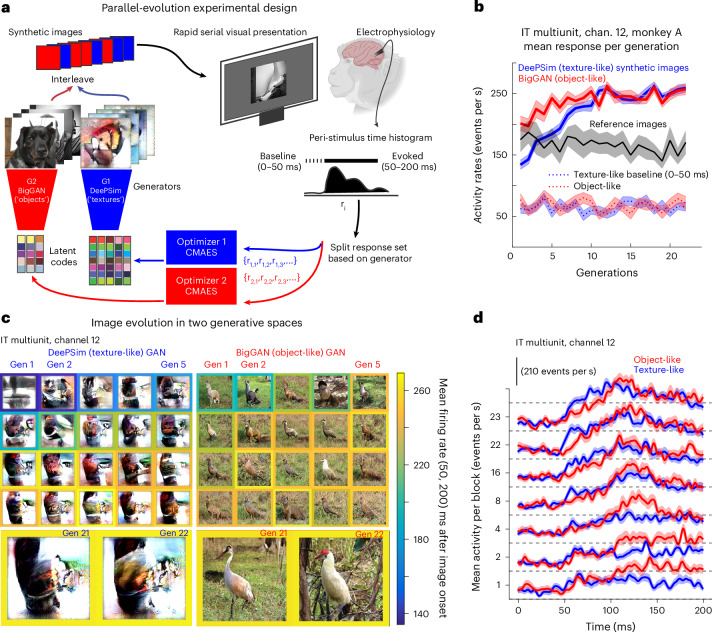
Example of a successful paired evolution from an inferotemporal cortex site. **a**, Schematic of the parallel evolution design. Synthetic images from DeePSim (texture-like) and BigGAN (object-like) were interleaved and optimized independently in a closed loop using CMA-ES. **b**, Mean firing rate per generation for one PIT multi-unit. Lines show means; shading, s.e.m. Red and blue traces show responses to BigGAN and DeePSim images; gray trace shows reference images; dotted lines indicate baseline (0–50 ms). **c**, Example images from successive generations in each space. Frames are color-coded by the mean evoked firing rate per block. **d**, PSTHs across generations for the same site. Lines show means across images in each block; shading, s.e.m. Source data

In each generation/block, images proposed by both threads were randomly interleaved to control for recording quality and behavioral state. Each image was shown once for 100 ms on and 150 ms off, with three to five images per trial and with fixation required within a 1°-radius window. After presentation, the mean firing rate (50–200 ms window) for each image was used as a scalar score for the optimizers, which then proposed new latent vectors for the next block (Fig. 2a). We collected 170 paired evolutions and retained 154 sessions (90 from monkey A, 64 from monkey B) after excluding unstable or short sessions (<15 blocks).

In a representative PIT multi-unit experiment from monkey A, both texture-based and object-based algorithms increased firing rates (Fig. 2b). Optimized images showed different global configurations but shared local motifs. DeePSim began from amorphous textures, while BigGAN began with a well-formed alpaca-like shape. After convergence, the driver unit guided DeePSim toward a brown curved surface and BigGAN toward a bird-like creature on grass (Fig. 2c). Below, we will show that in general, neurons guided both generators to synthesize local features, such as the curved edge present in the bird’s neck, well-isolated in the DeePSim evolution. Despite their global dissimilarity, both sets evoked comparable firing rates (mean firing rate for images of the last two blocks; *t*-test *t*_128_ = 0.97*, P* = 0.33), suggesting that the neuron responded similarly when key local features were present. Peri-stimulus time histograms (PSTHs) revealed longer peak response latencies to the object than texture images (Fig. 2d), quantified across the population in an upcoming section.

### Image similarity was local and related to response dynamics

**Fig. 3:**
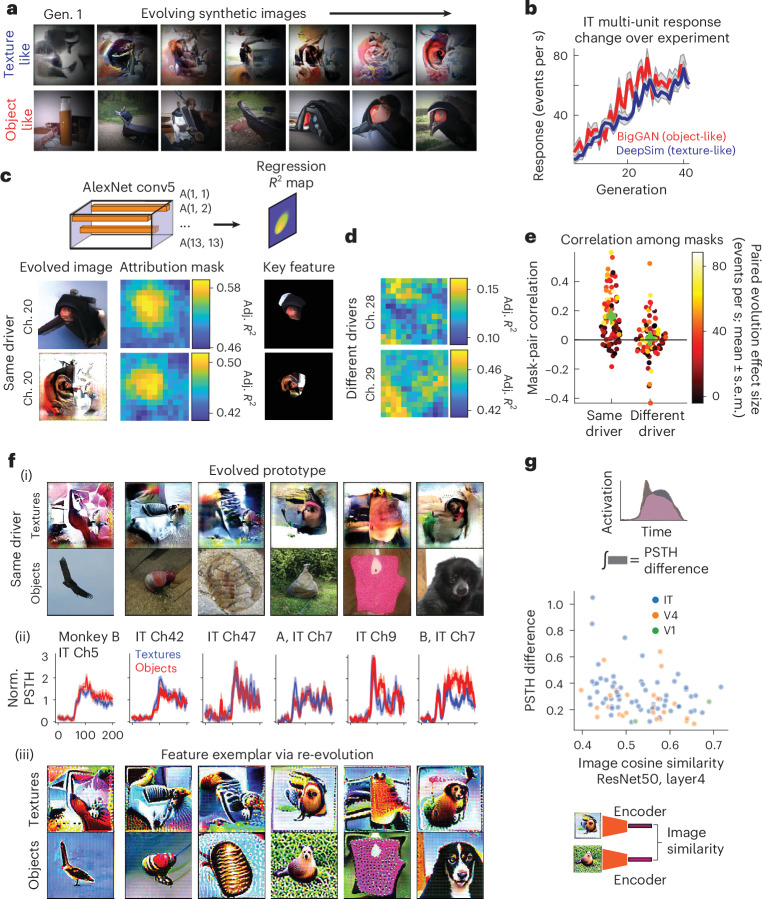
Optimized images showed local feature similarity. **a**, Example paired evolution from a PIT multi-unit (monkey B, channel 20). Synthetic images evolved over generations in DeePSim (texture-like) and BigGAN (object-like) spaces. **b**, Mean firing rate per generation for the same site during texture (blue) and object (red) evolutions. Shading, s.e.m. **c**,**d**, Attribution masks were derived by regressing neuronal responses onto spatial CNN features (AlexNet conv5), with mask intensity indicating local adjusted *R*^2^; example masks are shown for the same driver site in **c** and for different drivers in **d**. **e**, Attribution-mask correlations were higher for the same driver than for different drivers. **f**, (i) Additional examples of evolved images. (ii) PSTHs for the most activating blocks (mean ± s.e.m.). (iii) Re-optimized ‘feature exemplars’ from computational models of the recorded evolutions. **g**, Image similarity (ResNet50 layer 4 embeddings) was inversely correlated with PSTH differences across paired evolutions (Pearson’s *r* = −0.29, *P* = 6.0 × 10^−^^3^, *n* = 87, two-sided). Source data

Individual neuronal sites could drive the evolution of synthetic images using two generators (texture-based DeePSim and object-based BigGAN). Visual inspection suggested that the same neurons produced globally distinct but locally similar images. For instance, IT site 20 in monkey B evolved two distinct images (Fig. 3a): the BigGAN image resembled a dark, rounded object with an orange-red center, while the DeePSim image was more of a pastiche of colors yet contained a similar orange-red feature in the same location. Both were equally activating (Fig. 3b). This raised two possibilities: either this site represented unrelated images (as in superposition and polysemanticity^35^) or it focused on shared local regions^36^.

**Extended Data Fig. 3:**
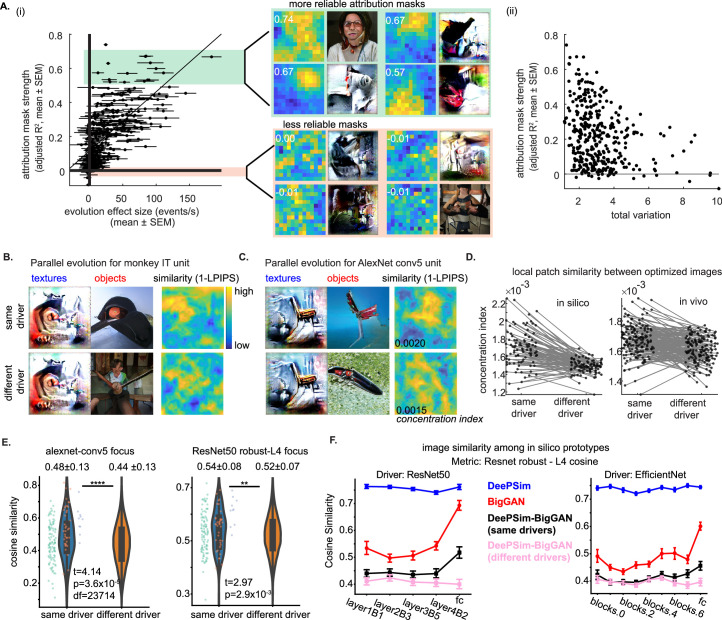
Spatial attribution map analyses. **A (i)** We measured the mean adjusted R^2^ for each map and observed that it positively correlated with the overall change in neural firing rates during the optimization experiment: the stronger the change in activity, the more reliable the spatial attribution mask was in capturing relevant image features. Specifically, across these experiments, we observed a range of changes in firing rate (events/s) from −12.1 to 183.1, with an overall mean of 25.3±1.7 (mean, SEM). Similarly, attribution mask R^2^ values ranged from −0.08 to 0.74, with an overall mean of 0.20±0.01 (mean, SEM). Not surprisingly, there was a strong correlation between evolution effect size and mean attribution mask R^2^ (Pearson correlation coefficient =0.672, P < 1×10−4). (ii) To measure whether evolution success affected the shape of the map, we used total variation (TV). TV serves to quantify the smoothness of a matrix by pooling the magnitude of gradients across the matrix surface (that is, the sum of the Euclidean norms of the gradient vectors at each matrix point). High TV suggests a noisier map. The median value for TV was 2.79 (inter quartile range: 2.16−3.61), while for the adjusted R^2^, it was 0.21 (0.07−0.32). They showed a Pearson correlation of −0.30 (P = 3.4×10^−9^). These results suggest that spatial masks were smoother as neural responses to optimized images became more robust. We also quantified correlations in linear weights derived from individual fits to evolutions for the same driving channel and compared them to correlations derived from fits to evolutions across randomly selected channels (non-drivers). We relied on the attribution mask, extracting and averaging weights from the largest connected region of high-goodness values. These weights were then used to compute correlations within the same driver condition and between randomly selected non-driver channels. We found that correlations in coefficient fits for the same driver were stronger than those in the random driver condition. The median correlation for the same driver condition was 0.034 ± 0.016 (SE) with *N* = 96 unique monkey channels (all animals), compared to 0.003 ± 0.003 (SE) with *N* = 1920 for the random driver evolutions; these values were statistically different per a Wilcoxon rank sum test (*P* = 5.6 × 10^−4^, Cliff’s delta of 0.208, indicating a small to moderate effect). **B**. **We used an** **image similarity metric (LPIPS) to compare optimized images**. Specifically, we used the LPIPS library to create a similarity map between the images (similarity =1− LPIPS distance). We saw that the similarity map was not homogeneous but acquired high values in local regions. To determine if these local regions were likely to arise by chance across unrelated images, we measured the similarity maps between BigGAN – DeePSim image pairs optimized for the same unit (same-driver maps, top row) and compared them against the heatmaps between BigGAN – DeePSim image pairs optimized for different units (different-driver maps, bottom row). **C. LPIPS similarity map between optimized images for the same in silico units** (AlexNet conv5) vs. optimized images for different units, along with an index showing local similarity concentration. This was based on a concentration index, a metric that measured the local concentration of activity by convolving the map with local Gaussian filters with diameters less than the full image. We first developed the concentration index in silico, by running parallel evolutions using AlexNet conv5 units, and found that the local concentration index measured 0.0017±0.0002 (mean ± SEM) between DeePSim-BigGAN images optimized by the same site, vs. 0.0015±0.00008 between images optimized by different units (P =7.4×10−14, Z-Val: 7.5, N =91, Wilcoxon signed-rank test, left). **D. Local patch similarity between optimized images**. Concentration index values comparing DeePSim–BigGAN image pairs optimized for the same driving site (“same driver”) versus image pairs optimized for different driving sites (“different driver”). Left: results from CNN units (in silico). Right: results from neuronal sites in V1, V4, and IT (in vivo) from monkeys A and B. Each dot corresponds to one pairwise comparison derived from a single driving site (biological replicates for the in vivo panel; independent units for the *in silico* panel). Lines connect paired comparisons within a site. For the *in vivo* dataset, the mean concentration index was 0.0017 ± 0.00010 (mean ± SEM) for same-driver image pairs and 0.0016 ± 0.00008 for different-driver image pairs (N = 163 sites; Wilcoxon signed-rank test, P = 3.2 × 10⁻⁸, Z = 5.5). This indicates greater local patch similarity between DeePSim–BigGAN images optimized from the same neuronal driver compared to images drawn from different drivers. **E. Image similarity measured by other neural networks**. The violin plot shows cosine similarity between feature exemplars generated by the same driver neuron/multiunit (“same driver”) versus different neurons/multiunits (“different drivers”); while the exemplars are biological, the comparisons are made in the representational space of different networks. **In all networks tested, exemplars from the same driver neuron were significantly more similar than those from different neurons:** AlexNet conv4 focus (Same: 0.609 ± 0.098, Unpaired: 0.578 ± 0.096, *t* = 3.93, *p* = 8.5 × 10^−5^), VGG conv4 focus (Same: 0.715 ± 0.082, Unpaired: 0.695 ± 0.091, *t* = 2.73, *p* = 6.4 × 10^−3^), ResNet50 robust L3 focus (Same: 0.815 ± 0.050, Unpaired: 0.805 ± 0.048, *t* = 2.37, *p* = 1.8 × 10^−2^), AlexNet conv5 focus (Same: 0.481 ± 0.130, Unpaired: 0.439 ± 0.126, *t* = 4.14, *p* = 3.7 × 10^−5^), VGG conv5 focus (Same: 0.453 ± 0.102, Unpaired: 0.432 ± 0.103, *t* = 2.52, *p* = 1.2 × 10^−2^), ResNet50 robust L4 focus (Same: 0.539 ± 0.076, Unpaired: 0.521 ± 0.073, *t* = 2.97, *p* = 2.9 × 10^−3^, tests were two-sided). For each layer, we averaged the activation tensor (weighted, along the spatial dimension), obtaining an embedding vector with the same dimension as the channel dimension. The spatial averaging weight was a simple matrix that highlighted the center of the feature map and gradually fell off to zero at the border. This weight was a heuristic to emphasize features that were spatially more aligned with the neuronal receptive field. All 154 experiments were included. Consistently across metrics, feature exemplars from DeePSim and BigGAN space were more similar when they were driven by the same neuronal site than when driven by different sites. Violin plots show the full distribution of values. Box plots inside violins indicate the median (center line), the 25th and 75th percentiles (lower and upper box edges), and whiskers extending to 1.5× the interquartile range. **F. Similarity of Prototypes in DeePSim and BigGAN Space for Units** ***in silico***. We conducted parallel evolutions on convolutional network units from ResNet50, ResNet50-robust, EfficientNet-B6, and EfficientNet-B6 AdvProp. From each network, 50 units were sampled from selected layers, and each unit was evolved 10 times in both DeePSim and BigGAN space. We then computed cosine similarities between prototypes evolved by the same hidden unit (within a generator or across generators) and between prototypes evolved by different units. This defined the expected similarity range from this approach, independently of neuronal response noise. The plots show mean cosine similarity ± SEM for four conditions: **DeePSim same-unit (cyan)**, **BigGAN same-unit (magenta)**, **cross-generator same-unit (black)**, and **cross-generator different-unit (blue)**. Within-generator, same-unit prototypes showed the highest similarity (especially in DeePSim), cross-generator prototypes of the same unit showed intermediate similarity, and different-unit comparisons were lowest. This hierarchy was consistent across networks, with deeper layers showing larger separations between same- and different-unit prototypes.

We investigated these alternatives by generating spatial attribution masks that localized image regions most associated with neural activity. Masks were derived by linearly regressing recorded neuronal responses onto deep features from the final convolutional layer (conv5) of AlexNet. For each spatial location in the 13 × 13 activation map, hidden unit activations defined the feature vector for each local image patch, and a linear model predicted neural responses across all evolved images, yielding spatial attribution based on adjusted *R*^2^ (Fig. 3c). Spatial masks were smoother and cohesive when evolutions were successful (Extended Data Fig. 3). We then measured correlations among attribution masks for DeePSim–BigGAN pairs from the same sites (*n* = 84) versus different sites. Same-site correlations ranged between −0.18 and 0.60 (median, 0.13 ± 0.029). By contrast, the mask correlations for different drivers ranged between −0.43 and 0.52 (median, 0.004 ± 0.008, *P* = 6.0 × 10^−7^, Wilcoxon signed-rank test). Additional perceptual similarity analyses^37^ (learned perceptual image patch similarity (LPIPS)) confirmed that same-site pairs shared concentrated local similarity patches rather than global overlap (Extended Data Fig. 3).

To identify preferred features, we fit predictive models to each evolution’s image-response data and re-optimized images in silico to generate ‘feature exemplars’ that emphasized salient features^38^ (Methods and Fig. 3f). We then tested whether paired evolution images were more similar by computing their feature-space similarity with a pretrained encoder. Feature-space similarity between maximal DeePSim and BigGAN images for the same unit was higher than between unmatched drivers (ResNet50-robust layer 4 embedding, paired vs unpaired, mean ± s.d., 0.414 ± 0.084, *n* = 154 vs 0.397 ± 0.078, *n* = 23,562; independent *t*-test, *t*_23714_ = 2.61, *P* = 9.1 × 10^−3^). Similarity increased for re-evolved exemplars (paired vs unpaired, 0.539 ± 0.076 (*n* = 154) vs 0.521 ± 0.073 (*n* = 23,562); *t*_23714_ = 2.97, *P* = 2.9 × 10^−3^; Extended Data Fig. 3e), with consistent results across encoders (for example, AlexNet, VGG). Parallel in silico analyses showed the same pattern: dual-evolution images generated by the same hidden unit were more similar than those from different units (Extended Data Fig. 2d).

Finally, we asked how image similarity related to neuronal dynamics. We computed the mean PSTH during the maximum activation block in each generative space and compared these paired PSTHs. The integrated absolute difference between the two normalized paired PSTHs (*d*_PSTH_) predicted image similarity (*c*_embed_), with a Pearson correlation of −0.293 (*n* = 87, *P*= 0.006 for successful evolutions; Fig. 3g). In this analysis, the PSTH distance was a better predictor of image dissimilarity than the difference in mean firing rate *d*_act_ (−0.098, *n* = 87, *P* = 0.37 (NS)). Thus, the temporal response structure carried information about cross-generator image similarity.

Together, these results indicate that neurons guided image synthesis in two distinct latent spaces that nonetheless shared local visual features. Although superposition may still occur in certain temporal windows, these results show that neurons could adapt to both spaces when overlapping local features were present.

### Alignment as a facility of hill climbing

Here, we examine how each generative latent space aligned with neuronal tuning. We reasoned that because the optimizer operates in the GAN latent space, the neuronal tuning function with respect to the latent variables can be conceptualized as an energy landscape; a smoother landscape would yield easier optimizations: higher success rates, faster convergence and greater peak activation. We quantified alignment using three complementary metrics: facility of hill climbing, reflecting optimization success; the climb’s start and endpoint, defined by activations to unoptimized and optimized images; and climb speed, indicated by convergence rate. Mathematical treatments of the concepts Alignment and Manifold appear in the Extended Data.

**Fig. 4:**
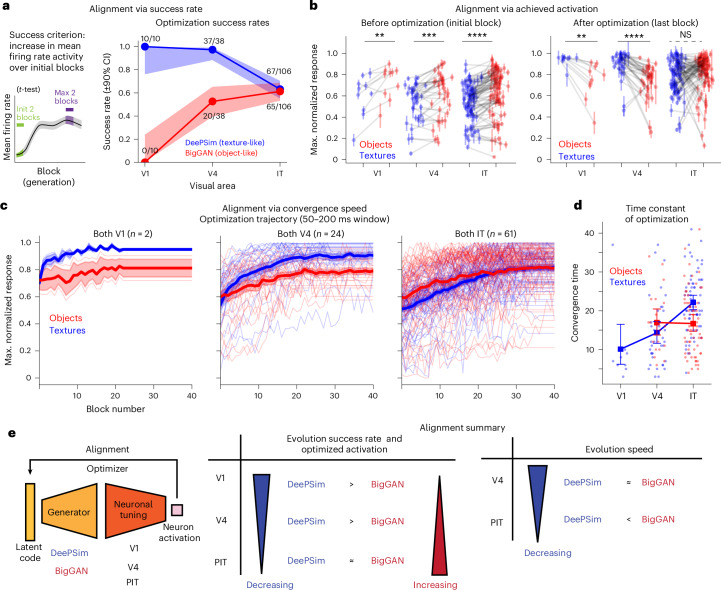
Differential alignment with DeePSim and BigGAN across the ventral hierarchy. **a**, Alignment measured by evolution success rate. Success was defined as a significant increase in firing rate relative to the initial two blocks (*t*-test, *P* < 0.01, two-sided, same below). Numbers indicate successful sessions per area and generator. **b**, Alignment measured by achieved activation. Normalized neuronal activity before (left; initial block) and after optimization (right; final block). For each paired evolution, firing rates were averaged across trials within each block and normalized by the maximum block-wise mean across all blocks and both threads (the same normalization in **c** and **d**). Red points indicate object-optimized threads and blue points indicate texture-optimized threads (error bars denote mean ± s.e.m within block); gray lines connect threads in paired evolutions targeting the same neuronal site. Sessions were included if at least one thread met the success criterion. Differences between texture (DeePSim) and object (BigGAN)-optimization values were assessed using a two-sided paired *t-*test. For DeePSim > BigGAN, the test yielded the following values: before optimization: V1**:** ${t}_{9}=-3.49,\,P=6.8\times {10}^{-3}$; V4: ${t}_{36}=-4.25,\,P=1.4\times {10}^{-4}$; and IT: ${t}_{85}=-7.32,\,P=1.3\times {10}^{-10}$ after optimization: V1: ${t}_{9}=4.65,\,P=1.2\times {10}^{-3}$; V4: ${t}_{36}=5.99,\,P=7.3\times {10}^{-7}$; and IT: ${t}_{85}=-1.41,\,P=0.16$. Sample sizes (sessions in which at least one thread succeeded, *P* < 0.05) were V1, *n* = 10; V4, *n* = 37; and IT, *n* = 86. **P < 0.01; ***P < 0.001; ****P < 0.0001. **c**, Alignment measured by evolution trajectory. Normalized block-mean response trajectories or sessions in which both threads succeeded. Thin lines indicate the trajectory of individual threads; thick line indicates the mean across threads; shading indicates s.e.m. Sample sizes (sessions in which both threads succeeded, *P* < 0.05) were V1, *n* = 2; V4, *n* = 24; and IT, *n* = 61. **d**, Convergence time constants for successful threads. Time constants were estimated from normalized response trajectories for successful threads. Small dots represent individual evolution threads, and large symbols denote the mean and 95% CI of the mean. Sample sizes (successful threads, unpaired, *P* < 0.01) were DeePSim (blue dots): V1, *n* = 10; V4, *n* = 37; and IT, *n* = 67; BigGAN (red dots): V1, *n* = 0; V4, *n* = 20; IT, *n* = 65. **e**, Schematic summary. Along the ventral hierarchy, neuronal alignment with DeePSim (blue) decreased while alignment with BigGAN (red) increased, converging at a similar level in PIT. Source data

We first asked how easily neurons in each visual area could guide the evolution process on each image manifold, quantifying this through the success rate across evolution experiments. An evolution was deemed successful if neuronal activity in two consecutive blocks exceeded that of the first two blocks (50–200 ms window, 25–40 images per block, Student’s *t*-test). Using this criterion (*P* < 0.01), the overall success rate of BigGAN evolutions (55.2%; 95% CI (48.6%, 61.6%)) was lower than that of DeePSim (74.0%; 95% CI (67.8%, 79.3)). Under a more lenient criterion (*P* < 0.05), rates were comparable: DeePSim, 75.3%; 95% CI (69.1%, 80.5%) vs BigGAN, 67.5%; 95% CI (61.0%, 73.3%) (Extended Data Table 1). However, success patterns differed across areas. For object evolutions, the success rate increased from V1 (0 out of 10), through V4 (20 out of 38) and PIT (61%; 65 out of 106). By contrast, for texture evolutions, the success rate fell from V1 (100%; 10 out of 10) through V4 (37 out of 38) to PIT (67 out of 106; Fig. 4a). These trends persisted across alternative metrics (Extended Data Table 1). Thus, texture and object spaces diverged sharply in V1 and V4 but showed comparable success in PIT.

In PIT, evolution success in one space correlated with success in the other (*χ*^2^_1_ = 18.55, *P* = 1.7 × 10^−5^), suggesting that common factors such as responsiveness or signal quality partially determined success in both spaces. Overall, visual cortex neurons could guide different image generators to produce highly activating stimuli, consistent with smooth, continuous tuning on both image manifolds that allowed optimizers to ‘climb’ slopes in each space. The differences across the hierarchy indicated stronger alignment of texture parametrization in V1 and V4, and comparable alignment of object and texture parametrizations in PIT (Fig. 4e).

### Alignment as the starting and ending points of hill climbing

Next, we examined how each generator’s priors aligned with different areas before optimization. Specifically, we asked whether neuronal responses to the earliest generations from the texture and object generators differed, and if so, in what visual area. Spike rates were normalized (*z*-scores) and grouped by generator type (DeePSim or BigGAN). In the first generation, BigGAN generally evoked higher activations across all areas (Fig. 4b; BigGAN > DeePSim, paired *t*-test, max > initial, *P* < 0.05, *t*_9_ = −3.491, *P* = 6.8 × 10^−3^ for V1, *t*_37_ = −4.399, *P* = 8.9 × 10^−5^ for V4, *t*_105_ = −9.286, *P* = 2.4 × 10^−15^ for PIT, *n* = 154 experiments). However, V1 and V4 neurons rapidly climbed in texture space, and within the first few generations, their responses matched: for the first four blocks, we found no significant differences in median responses to early images from V1 (*z*-score, median ± s.e.m.: BigGAN = −0.09 ± 0.11, DeePSim = −0.12 ± 0.09; *P* = 0.84, Wilcoxon rank sum test, rank-biserial correlation = 1.15, *n* = 11 paired values) or from V4 (BigGAN = −0.23 ± 0.09, DeePSim = −0.25 ± 0.08; *P* = 0.73, rank-biserial correlation = 1.10, *n* = 25 paired values). By contrast, PIT responses diverged (BigGAN = −0.04 ± 0.09, DeePSim = −0.34 ± 0.06; *P* *<* 0.002, rank-biserial correlation = 1.49, *n* = 60 paired values). Collectively, the default visual statistics learned by BigGAN were well-suited for visual neurons, compared to the initial textures learned by DeePSim, but this was more reliably seen for PIT neurons.

After optimization, the results shifted. For experiments in which at least one thread succeeded, texture images evoked higher activations for V1 and V4 neurons (*t*_9_ = 4.651, *P* = 1.2 × 10^−3^ for V1 and *t*_36_ = 5.985, *P* = 7.3 × 10^−7^ for V4); whereas PIT neurons showed similar activation levels (*t*_85_ = −1.41, *P* = 0.16 (NS)); in monkey A, BigGAN slightly exceeded DeePSim (*t*_47_ = −2.32, *P* = 0.025) and in monkey B, activations were comparable *t*_37_ = 0.70, *P* = 0.49; Fig. 4b). Restricting analyses to experiments in which both threads succeeded (*n* = 87), V4 neurons still favored textures (DeePSim, mean ± s.e.m., 0.898 ± 0.028; BigGAN, 0.784 ± 0.020, *t*_23_ = 3.707, *P* = 1.2 × 10^−3^); while PIT activations remained comparable (DeePSim, 0.821 ± 0.018; BigGAN, 0.820 ± 0.021, *t*_60_ = 0.06*, P* = 0.95). Hence, as in success rate analyses, PIT closed the optimization gap between textures and objects.

In silico evolutions revealed a contrasting trend: all layers favored texture-based optimizations, highlighting a gap between artificial and biological representations (Extended Data Fig. 2). Nevertheless, as in visual cortex, we found that this activation gap shrank with network depth; in ResNet50-robust, units in the final object classification layer (fc) and the penultimate layer (block4B2) showed smaller gaps between the DeePSim–BigGAN activations than earlier layers (two-sample *t*-test, DeePSim–BigGAN gap in blocks 1,2,3,4 > gap in fc, *t*_998_ > 16, *P* < 5 × 10^−55^, for all earlier blocks).

In summary, neuronal optimization outcomes varied by area and image space. V1 and V4 neurons favored texture-based parametrizations, whereas PIT neurons aligned comparably with both texture and object manifolds. This dual alignment in PIT created a sharp contrast with CNNs (even at classification layers), highlighting a discrepancy between the visual stream and current computational models.

### Alignment as the speed of hill climbing

We next examined the optimization trajectory of neurons, or how neuronal activation changed across the evolution process. The geometry of a neuron’s tuning landscape may vary across manifolds (Fig. 1c), affecting optimization dynamics. We reasoned that if a manifold efficiently expressed the features favored by a neuron (if the space aligned better with it), then the evolution should converge faster in that space. We computed average optimization trajectories per area (Fig. 4c). For V1 and V4 neurons, although object-space activations started higher, texture space activations quickly surpassed them. By contrast, for PIT neurons, object space remained higher throughout and reached similar final activation as texture space.

We quantified the win rate of each space during evolution, defined as the fraction of sessions for which responses to BigGAN exceeded those to DeePSim images (Student’s *t*-test), or vice versa, for a given generation. For V1 and V4, BigGAN initially won about 40% sessions; but only after two or three blocks, its win rate dropped to near zero, while ~60% of DeePSim threads climbed higher. For PIT, more than 40% of experiments maintained higher BigGAN activations throughout. Parallel analyses of CNN units showed similar trends: deeper CNN layers required more iterations for DeePSim activations to surpass BigGAN’s (Extended Data Fig. 2).

In prior work using only the texture generator, higher visual areas took longer to optimize^18,19^, consistent with sharper, higher-dimensional tuning. Here, we quantified convergence time for both generators by measuring the number of generations required to reach 80% of the maximum activation increase (Fig. 4d). In DeePSim space, convergence time increased along the hierarchy (PIT > V4, 22.1 ± 1.0 (*n* = 67) vs 14.3 ± 1.4 (*n* = 37), *t*_102_ = −4.67, *P* = 9.3 × 10^−6^; PIT > V1, 10.1 ± 3.1 (*n* = 10), *t*_75_ = −4.40, *P* = 3.6 × 10^−5^). In BigGAN space, no such trend was observed from V4 to IT. When both threads succeeded, PIT neurons converged faster in BigGAN space than in DeePSim (DeePSim > BigGAN, 22.3 ± 1.1 vs 17.7 ± 1.1 (*n* = 52), *t*_51_ = 3.36, *P* = 1.5 × 10^−3^). Alternative convergence measures yielded similar results (Extended Data Table 2).

Overall, these results suggest stronger alignment of PIT neuronal tuning to the object manifold than V1 or V4, while the continued effectiveness of the texture manifold indicates that BigGAN is not uniquely preferred by higher visual neurons.

### Object space aligned best to late responses in PIT

We have shown that along the ventral stream, neurons show increasing alignment with the object-based BigGAN space, reaching comparable alignment in PIT neurons, while CNN units continued to prefer texture-based DeePSim space. To explain this difference, we examined a feature that biological neurons possess but most vision models lack: dynamics. Visual neurons show time-varying responses to static images, reflecting shifts in encoded features^39–44^ from broader to sharper tuning, from local to holistic or coarse to fine-grained information. We analyzed these dynamics using PSTHs from each evolution. By design, the optimization objective was to increase firing rate in the 50–200 ms window, but it was up to the neurons whether this increase arose from early-transient, late-sustained or off responses (or all together). We analyzed the mean response after image onset (the PSTH, response as a function of time in milliseconds) and also the time-binned mean response over the image optimization process (response in short-time windows, as a function of block/generation during the evolution).

**Fig. 5:**
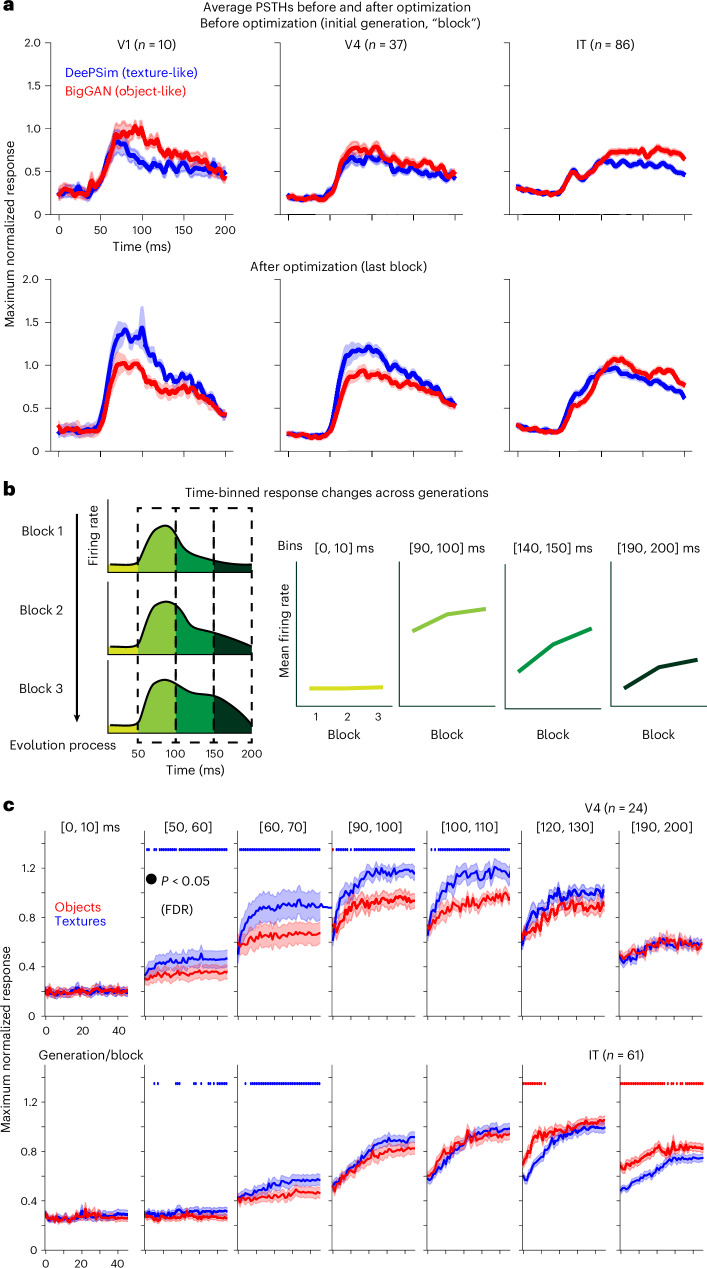
Object space preferentially activated late responses in PIT neurons. **a**, Population-averaged PSTHs from V1, V4 and PIT before (top) and after (bottom) optimization. Thick lines indicate means across threads; shading, s.e.m. Sample sizes (sessions in which at least one thread succeeded, *P* < 0.05) were: V1, *n* = 10; V4, *n* = 37; and IT, *n* = 86. **b**, Schematic of the time-binned analysis. Evoked firing rates were computed in fine (10 ms) windows and tracked across generations to obtain temporal evolution trajectories. **c**, Evolution trajectories of V4 and PIT neurons for representative 10 ms time bins (mean ± s.e.m.). In PIT, texture-based evolutions (blue) dominated early responses, whereas object-based evolutions (red) prevailed in later windows. Filled circles denote blocks with significant differences after false discovery rate (FDR) correction (paired *t*-test, two-sided). Sample sizes (sessions in which both threads succeeded, *P* < 0.05) were V4, *n* = 24; IT, *n* = 61. Source data

**Extended Data Fig. 4:**
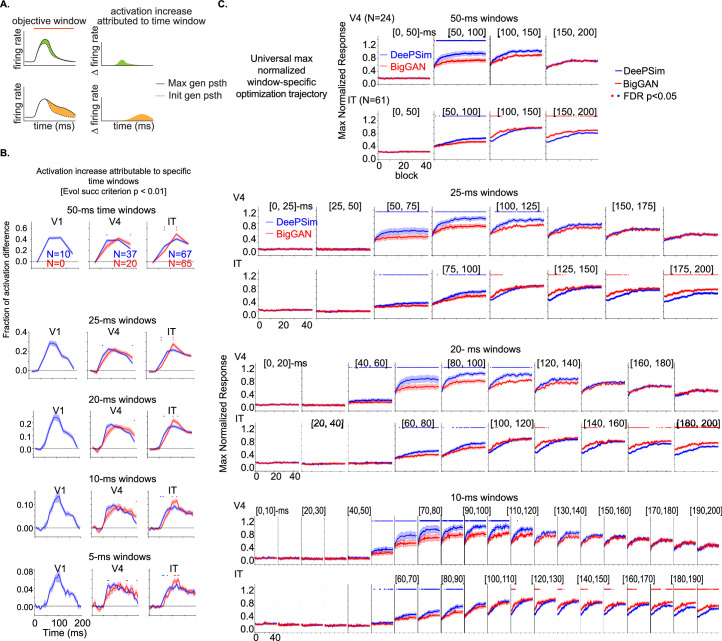
Extended visualization of neuronal dynamics analysis during evolution. **A**. Schematics of the temporal attribution analysis, activation difference between PSTHs of the first and max activation block is normalized and attributed to each time bin. **B**. Difference in temporal attribution strength between DeePSim- and BigGAN evolution threads computed in time bins ranging from 5 ms to 50 ms, threads with significant activation increase were included, non-paired. Error bands represent mean ± SEM across evolution threads. Independent t-test (two-sided) were applied separately at each time bin, with false discovery rate (FDR) correction applied to the raw p values across all time bins to control for multiple comparison. Statistical significance was defined at FDR corrected p < 0.05. Dots on top row indicate significant comparisons per FDR, dots on lower row indicate significance per original p < 0.05; Red dots indicate time bins where BigGAN > DeePSim, and blue dots indicate DeePSim > BigGAN. Exact P values for significant bins ranged from p = 0.0037 to p = 0.042 (the full sets of p values can be reproduced via released code and data). Sample size (number of successful evolution threads, p<0.01) was DeePSim: V1, N = 10; V4, N = 37; IT, N = 67; BigGAN: V1, N = 0; V4, N = 20; IT, N = 65. **C**. Normalized evolution trajectories for specific time windows using time bins of 10, 20, 25, and 50 ms (similar to Fig. 5c), same normalizer was applied for each paired evolution across the two threads and across block and time windows. The line and shaded error bar indicates mean ± SEM across threads. At each block, activations from DeePSim and BigGAN evolution trajectories were compared using a paired t test (two-sided). Raw p values from each block underwent False Discovery Rate (FDR) correction, and statistical significance was determined at corrected p < 0.05, and significant blocks are indicated by colored dots (the full sets of p values can be reproduced via released code and data). Sample size was N=24 neuronal sites in V4, N=61 in IT, for all analyses. Source data

Average PSTHs differed across both spaces before and after optimization (Fig. 5a). We quantified activation increases within different time bins after image onset. For all successful evolutions (*P* < 0.05, two-sample *t*-test), we expressed the firing rate change in each 10 ms time bin as a fraction of the total (0–200 ms). In PIT, BigGAN-driven activation increases were greater at later times ((100, 110) ms, DeePSim > BigGAN, two-sample *t*-test, same below, *t*_130_ = −2.129, *P* = 0.035) and (120, 130) ms (*t*_130_ = −2.967, *P* = 0.0036) and smaller at earlier times ((50, 60) ms (*t*_130_ = 2.144, *P* = 0.034) and (60, 70) ms (*t*_130_ = 2.574, *P* = 0.011) (Extended Data Fig. 4b). Thus, successful BigGAN evolutions preferentially recruited later PIT responses.

We next examined how PSTH dynamics interacted with optimization over time. We computed firing rates in 10 ms bins and tracked them across session blocks as a time-split optimization trajectory (Fig. 5b). For V4, responses to DeePSim images exceeded those to BigGAN images from 50 ms to 110 ms (Fig. 5c, upper) and were comparable later. However, for PIT, DeePSim dominated early ((50, 90) ms), but BigGAN surpassed it from 110 ms onward (Fig. 5c; full plots, Extended Data Fig. 4c).

In summary, although time-averaged activity after optimization was comparable across spaces for PIT neurons, their dynamics diverged: object images evoked and recruited stronger late-stage activity, whereas V4 responses showed no temporal difference.

### Charting tuning landscapes in the BigGAN latent space

**Fig. 6:**
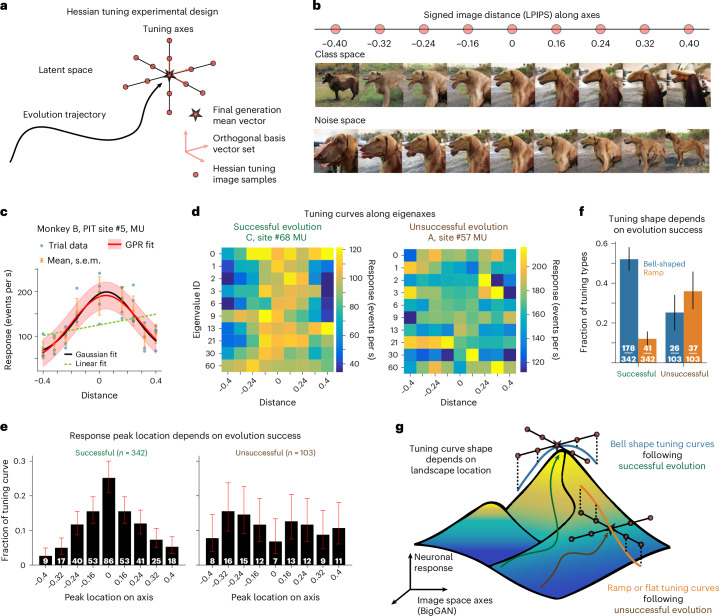
Geometry of tuning landscapes in BigGAN latent space. **a**, Hessian tuning design. After evolution, tuning was sampled along orthogonal axes in the BigGAN latent space originating from the final mean vector (red star). Firing rates were measured for images generated at incremental latent space steps. **b**, Signed image distances (LPIPS) along two example axes (class and noise) illustrate systematic changes in image content with controlled distance values. **c**, Example PIT site (#5, monkey B, multi-unit (MU)) showing bell-shaped tuning along a sampled axis. Responses (mean ± s.e.m.) plotted against signed image distance were fit with Gaussian (black) and linear (green) tuning functions. Red curve shows the Gaussian-process regression (GPR). **d**, Neuronal responses along different eigen-axes in class space; for example, driver sites, showing structured tuning across eigenvector dimensions (one eigen-axis per row, same signed image distance per column). Left: following successful BigGAN evolution (#68, monkey C); right: following unsuccessful ones (#57, monkey A). **e**, Distribution of tuning curve peak locations. Tuning curves following successful evolutions have peaks at (or close to) the center of the sampled axes, while those after unsuccessful evolutions have peaks distributed across the axes. Bars indicate the fraction of tuning curves peaking at each location (summing to 1), with error bars showing Wilson binomial confidence intervals. Sample sizes (number of significantly modulated tuning curves, *P* < 0.01): successful group, *n* = 342; unsuccessful group, *n* = 103. **f**, Distribution of tuning curve types. Fraction of significantly modulated tuning curves (per ANOVA) classified as bell-shaped (blue) or ramp-shaped (orange) for neuronal sites, conditioned on the preceding evolution experiment successfully having increased firing rate. Each bar represents the fraction of tuning curves, and error bars reflect beta-distribution-based confidence intervals. Sample sizes (number of significantly modulated tuning curves, *P* < 0.01): successful group *n* = 342; unsuccessful group *n* = 103. **g**, Conceptual illustration showing how sampling range within image space influences apparent tuning shape. Source data

To examine the shape of tuning functions in object space, we conducted new experiments using the original two monkeys (A, B) and an additional two (C, D). In earlier work, we mapped neuronal responses over two-dimensional surfaces within the DeePSim texture space and termed these functions tuning landscapes^19^. When neuronal activity was driven to high levels by optimized images, neurons showed smooth, bell-shaped tuning around those peaks, contrasting with reports of ramp-shaped tuning in face neurons^45,46^. We speculated that on the tuning landscape defined over a large image manifold, such as that of BigGAN, one could trace multiple one-dimensional tuning shapes. To establish the geometry of neuronal tuning in BigGAN, we performed Hessian tuning experiments: after completing optimization in object space, we started from the endpoint in that latent space trajectory and explored along 10–20 orthogonal axes adaptively chosen in that space, measuring firing rate changes along each one-dimensional axis (Fig. 6a). Mathematically, this quantifies:

$$r(\alpha )=f(G({z}^{\ast }+\alpha {\nu }_{i}))$$

where $G$ is the BigGAN generator, ${z}^{* }$ is the final latent vector, ${v}_{i}$ is an orthogonal basis vector and $\alpha$ is the step size. Step size was tuned by line search so that image changes (per LPIPS^37^) were comparable across axes (Fig. 6b). This approach revealed local tuning geometry and, in particular, the first-order or second-order differentials. Returning to the hill-climbing analogy: when neurons reached a local maximum, movement along a linear axis typically yielded a bell-shaped curve (Fig. 6c,d). However, if optimization ended far from a peak, curves appeared ramp-shaped (Fig. 6d). We conducted 55 Hessian tuning experiments in total (using the BigGAN latent space), excluding early pilots, and analyzed 1,088 tuning axes. A one-way ANOVA test revealed that 445 of these axes were significantly modulated by image changes ($P < 0.01$). The final activation reached during the BigGAN evolution strongly influenced Hessian tuning outcomes. When evolutions increased firing rates strongly, 51.8% (342 out of 660) of axes also showed significant tuning ($P < 0.01$, ANOVA, mean *F*-ratio = 4.81). By contrast, when firing rates were weaker, only 23.6% (103 out of 436) of the axes showed tuning (mean *F*-ratio = 2.18).

#### Peak locations

Peak positions depended on evolution success. In evolutions with strong activity, peaks clustered near the axis center (*d* = 0), with a mean peak distance of 0.178 ± 0.007 (*n* = 342) (Fig. 6e). In weaker cases, peaks were more dispersed, with a mean peak distance of 0.253 ± 0.011 (*n* = 103). A Wilcoxon signed-rank test showed this difference was significant (*z* = −5.26, *P* = 1.4 × 10^−7^, two-sided).

#### Tuning curve shapes

The shape of tuning curves also depended on evolution success. When evolutions reached high levels, among significantly modulated axes, 52% (178 out of 342) were bell-shaped, while 12% (41 out of 342) were ramp-shaped (monotonic). By contrast, when the evolutions did not reach high levels, only 25% (26 out of 103) of tuning curves were bell-shaped, while 36% (37 out of 103) were ramp-shaped. Two-proportion *z*-tests confirmed differences between the successful and unsuccessful cases for bell-shaped (*z* = 4.79, *P* = 8.5 × 10^−7^) and ramp-shaped curves (*z* = −5.60, *P* = 1.1 × 10^−8^; Fig. 6f). These findings indicate that ventral stream neurons can exhibit bell-shaped or ramp-shaped tuning, depending on where stimulus images reside relative to the neurons’ activity peaks (Fig. 6g).

## Discussion

### Summary of results

Using advances in deep generative models and evolutionary algorithms, we showed that neurons in the primate ventral stream can successfully guide image optimizations (evolutions) in two generative image spaces: a texture-based space (DeePSim) and an object-based space (BigGAN). Even when parallel evolution achieved comparable neuronal activations, they produced globally distinct images with shared local motifs. The two spaces differed in several optimization aspects, complementing prior single-generator studies^18,19^. Ascending the ventral hierarchy, the texture-based generator showed declining success rates and slower convergence, whereas the object-centric, photorealistic generator exhibited higher success rates and relatively fast convergence. In other words, optimization became more difficult in the texture space but easier in the object space, converging to a comparable level.

### Broader theoretical implications

**Extended Data Fig. 5:**
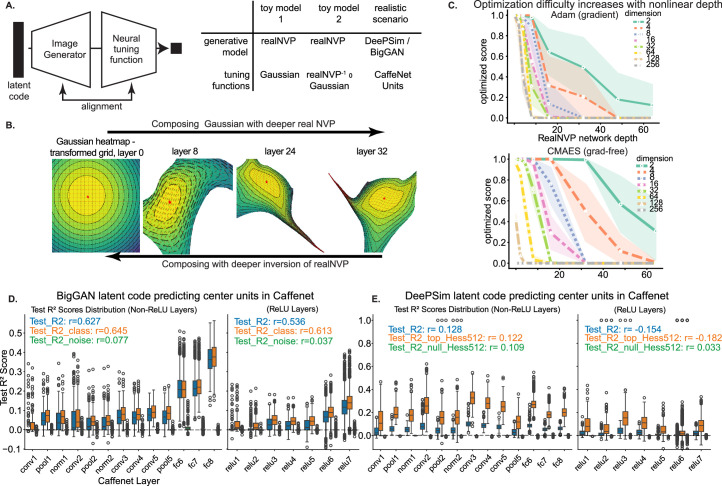
Mathematical treatment of alignment between generator and neuronal tuning. **A**. Conceptual schematics illustrating our definition of alignment between a generator and neuronal tuning, and a categorization of the three scenarios examined.**B**. Visualization of the transformed landscape of an isotropic Gaussian tuning curve when composed with deeper nonlinear (but invertible) networks (RealNVP). **C**. Optimization success (mean optimized score) as a function of space dimensionality and depth of nonlinear transformation, for gradient-based optimization (Adam, top) and evolutionary optimization (CMA-ES, bottom). Increasing the complexity of the nonlinear transformation (that is, greater depth of the invertible RealNVP network) lowers the best achievable activation level for the function with known optimal value (1.0). Shaded error bands show the mean and the 95% confidence interval of the mean estimated via bootstrapping. The sample size was 640 optimization runs (8 nonlinear depths × 8 dimensions × 10 random seeds per depth–dimension combination). **D**. Test of linearity for compositions of BigGAN with tuning functions of CaffeNet hidden units. For both non-rectified and rectified units, deeper units exhibit more linear relationships with BigGAN latent codes, as indicated by higher test ${R}^{2}$ values. This linearity is more pronounced for the class component of the latent code, whereas the noise component shows no significant linearity or hierarchical progression. Box plots indicate the median (center line), the 25th and 75th percentiles (lower and upper box edges), and whiskers extending to 1.5× the interquartile range or to the most extreme datapoint within that range. Outliers beyond the whiskers are shown as individual points. The sample size includes all 21,096 units in CaffeNet: 9,568 from rectified layers and 11,528 from non-rectified layers. **E**. Same analysis as in **D**, but using DeePSim as the generative model. Linearity between CaffeNet activations and DeePSim latent codes is less prominent and does not show a clear progression along the visual hierarchy. Within the latent code, most predictive power arises from the top Hessian eigenspace, while the bottom Hessian eigenspace contributes little predictive power. The sample size includes all 21,096 units in CaffeNet: 9,568 from rectified layers and 11,528 from non-rectified layers. Source data

**Extended Data Fig. 6:**
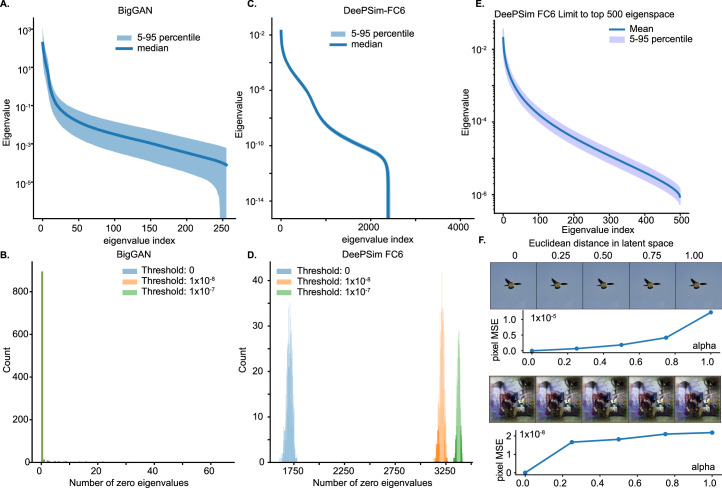
Empirical examination of the notion of a “manifold” in DeePSim and BigGAN latent spaces. We analyzed the spectrum of the Jacobian inner product (that is, the pullback metric / Hessian) of BigGAN and DeePSim-FC6, considering both the full latent space and the top-500-eigenvector subspace, to assess the extent to which these generators satisfy the conditions of a Riemannian manifold. **A, C, E**. Eigenvalue spectra of the Jacobian inner product. The center line indicates the median eigenvalue across 1,000 randomly sampled latent points, and the shaded region denotes the 5th–95th percentile range. **B, D**. Histograms showing the number of near-zero eigenvalues at each sampled latent vector (1,000 total). Different colors correspond to different eigenvalue thresholds used to define near-zero modes. **F**. Illustration of violations of the non-intersection (injectivity) property in DeePSim and BigGAN: traversing one unit along a null vector in latent space produces negligible pixel space mean squared error (MSE) and effectively no perceptual change in the generated image.

We propose the notion of alignment between neuronal tuning and generator mapping (detailed mathematical treatments of the concepts Alignment and Manifold appear in Extended Data Figs. 5 and 6). When neurons are smoothly tuned to the latent variables parametrizing images, the optimization becomes easier owing to smoother landscapes, empirically measured here. This supports the hypothesis that the higher visual cortex becomes increasingly able to invert mappings from latent variables related to objects while retaining the capacity to invert sub-semantic, texture-like variables. Such flexibility should make it easier for neurons in downstream circuits to encode the visual environment. This idea parallels prior findings in the face-processing system, whereby neuronal codes invert generative models of faces^47^; here, we extend that principle to broader image spaces. Our study also extends the concept of alignment past representational similarity between deep networks and neural codes^48–51^. Furthermore, we demonstrate a more causal sense of alignment: the ability of the neural code to steer latent variables of generative models.

### Connections to emerging questions in neuroscience

These dual optimizations raise broader questions about visual coding. Given that neurons could lead to optimized images with different visual priors, two explanations emerge: superposition, whereby units encode seemingly unrelated concepts^52^; or selectivity for simpler local features embedded in global configurations, as suggested by prior work^11,36^. Our results favor the latter, highlighting a local compositional code that flexibly recombines tokens across scenes. Still, alignment dynamics may reflect shifts in representational codes within broader networks; for example, supporting encoding of object identity alongside orthogonal information such as position, size or texture^53^. The observed ventral transition suggests that local signals are progressively refined into representations that can support object recognition but also textures, landscapes and navigational landmarks.

The finding that tuning curves can appear bell-shaped, ramp-like or even multi-peaked depending on optimization reinforces that neurons are not confined to single axes of selectivity, but operate in complex, high-dimensional tuning landscapes^19^. Such landscapes provide a geometric framework for understanding coding efficiency (as peak locations), invariance (as level sets^54^) and object recognition (as ensembles of peaks). This view helps explain why occipito-temporal neurons often respond to diverse, semantically unrelated images^55^: object selectivity arises from flexible recombination of local features rather than global templates. Within the broader tuning landscape, the untangling of object manifolds^56^ should therefore be viewed as one example of a general family of parallel operations that the system must perform for many feature domains, including space, textures, landmarks and more.

### Role of model complexity

BigGAN is architecturally more complex than DeePSim, raising an alternative explanation that results could stem from differences in steerability. This would predict uniformly stronger responses to DeePSim across areas, which we did not observe. Instead, we found a gradual shift favoring BigGAN along the ventral hierarchy, despite identical optimizer settings, indicating that the difference reflects neuronal tuning rather than algorithmic bias. Still, this architectural disparity is important. A limitation of this work is that DeePSim and BigGAN differ in architecture, training objective and feature representation, and we did not isolate which aspects most influence neural alignment. Future studies should dissect these factors systematically; for example, by varying network layers or training objectives one at a time, to identify the components most relevant to cortical responses.

### Relationship to previous work on texture versus object tuning

Our results also connect to studies examining the balance between texture-like and object-like representations in the visual cortex. We used the terms ‘texture’ and ‘object’ as accessible descriptors of the generators’ image priors (rather than as fixed categorical distinctions, as the ‘texture’ generator can be guided to produce more structured objects). Yet under the guidance of neuronal tuning, DeePSim tended to yield more texture-like stimuli, whereas BigGAN produced more object-like images. This links naturally to functional magnetic resonance imaging work showing that neurons in object-selective cortex can represent mid-level visual features^57^ and texture statistics as well as objects^58–60^, and our results would further support the view that the role of visual cortex is not to “explicitly encode objects but rather to provide a basis set of texture-like features” that can be used for multiple visual tasks^59^. We extend this conclusion by showing that this basis set includes spatially localized features, not only extended textures.

### Implications for computational modeling

No CNN aligned to BigGAN’s object space as closely as PIT did, indicating that current networks do not yet capture the full flexibility of the primate ventral stream. Such CNN–neural gaps create opportunities to strengthen benchmarking. Existing metrics such as Brain-Score^49,61^ assess alignment at the level of global representational similarity and should be complemented by more causal and local tests that break superficial correspondences. Physiologists might benefit from the development of deep models that flexibly align with both texture-like and object-like spaces comparably well, as the brain does.

### Implications for future neurophysiology

Each visual neuron might align best with a different image manifold, having its own energy landscape in image space. It is crucial to test whether more anterior regions in the ventral stream align even more closely with object-based BigGAN parametrizations.

### Principled criteria for selecting generative models in neuroscience

Future studies should extend this approach to diffusion models, which now dominate visual generative modeling. Diffusion models differ fundamentally from GANs, offering both opportunities and challenges for closed-loop neuroscience^62^. In addition to image quality, we should also consider additional desiderata such as the dimensionality of their latent or conditioning spaces, the match between their priors and known neuronal tuning and the smoothness of their latent-to-image mapping, which determines optimization navigability. Finally, this work underscores the importance of using multiple generators in closed-loop experiments. Using multiple generators guards against over-interpreting human-centric priors or architecture-specific artifacts^63^. This generative neuroscience approach offers new ways to probe neural representations while constraining the development of more biologically grounded models.

## Methods

### General setup

Experiments were controlled with MonkeyLogic2 (ref. ^64^) on ViewPixx EEG displays (120 Hz, 1920 × 1080 resolution). Viewing distance was 58 cm. Eye position was tracked with ISCAN. Animals fixated on a 0.25° target within a 1–2° window and received fluid rewards through a Crist DARIS module.

### Research animals

Two male rhesus macaques (A and B; 10–11 kg) were implanted with floating microelectrode arrays (Microprobes) in the right hemisphere V1–V2 border, V4 (prelunate gyrus) and PIT (anterior to the inferior occipital sulcus). Intraoperatively, array locations were based on sulcal landmarks, fine-tuned by local vasculature. For Hessian tuning experiments, the same procedures were repeated in two additional males (C and D; 14–15 kg). Arrays had between 16 and 32 working electrodes. All procedures were approved by the Institutional Animal Care and Use Committees at Harvard Medical School and Washington University and conformed to the Guide for the Care and Use of Laboratory Animals.

### Recording and preprocessing

Signals were acquired with Plexon OmniPlex. Sorting was performed online at session start to enable closed-loop operation. Signals within each channel were labeled 1–5, indicating confidence that activity reflected a single unit (1) versus multi-unit/hash (5), based on waveform shape, inter-spike interval and separation from the main hash signal. We use ‘unit’ or ‘site’ to refer to any sorted source. Most recordings used visually driven multi-units/hash and some used single units. After data collection, all spike/event times were discretized into 1 ms bins and convolved with a symmetric Gaussian probability density function with a 2 ms standard deviation. Sites were studied if visually responsive. For monkeys A and B, we recorded from 87 unique electrode locations; most of these sites (51) were sampled once, while the rest were sampled multiple times: 21 sites were sampled twice, six sites were sampled three times, four sites were sampled six times, two sites were sampled five times and one site was sampled nine times. There were an additional 160 unique sites from monkeys C and D, from which only a subset of channels were used. These sites show some correlation over time, although they cannot be designated rigorously as identical or fully independent neurons or neuronal populations. We generally used the Student’s *t*-test to perform two-sample tests of statistical significance (which assumes normality). Reported *P* values were calculated using two-sided tests, unless otherwise stated. Biological replicates were defined as individual neuronal recording sites in independent experimental sessions. Technical replicates (multiple trials within a block and repeated measurements across optimization blocks for the same neuron) were averaged or used for trajectory fitting and were not treated as independent samples. Each generator thread provided one measurement per neuron.

### Generative models

Two image generators were used.

The DeePSim *fc6* model^16^ was used with a custom MATLAB implementation translated from the original Caffe model and weights. This model comprises linear and up-convolution layers^25^, with a 4,096-dimensional latent space, within which around 500 dimensions are enough to capture the generated image variations^28^, with the rest effectively acting as a null space.

For BigGAN, we used the BigGAN-deep-256 version, implemented in the PyTorch-pretrained-BigGAN library. This model has a complex network structure, including self-attention^65^ and modulated batch normalization^66^. It is a class-conditional GAN, using a 128-dimensional class conditioning vector, $c\in C$ and a 128-dimensional noise vector, $z\in Z$. Each of 1,000 object classes in ImageNet is represented by a different class vector *c*, while the variations within each class are controlled by the noise vector $z$, sampled from a spherical truncated Gaussian distribution. Thus, sampling closely related points (‘traveling’) in the class embedding space $C$ interpolates between object categories, while traveling in the noise space $Z$ will change nuisance variables like aspect ratio, orientation or viewing angle^27,28^.

To quantify the nature of the priors within each generative space, we characterized the properties of the images produced by the generators. The FID^67^ indicated that BigGAN images were more ImageNet-like than DeePSim images. BigGAN sampled with class embeddings (50 images × 1,000 classes) yielded FID ≈ 10; BigGAN with Gaussian random class vectors yielded FID ≈ 44; DeePSim yielded FID ≈ 197. White and pink noise were much larger (≈ 416 and ≈ 380). Additional comparisons to COCO and THINGS showed intermediate FIDs (FID = 43.6 and 21.8). Low-level image features (luminance, contrast, color, sharpness, symmetry, entropy, edge density, frequency power) also differed between generators. Computational details and code libraries are provided in the Extended Methods section of Supplementary Information.

ImageNet photographs were also used as stimuli in multiple experiments, and some were instrumental in the results for Fig. 1a (*n* = 15 images) and Extended Data Fig. 1 (*n* = 5). Given copyright restrictions, these images were replaced with illustrative synthetic images, and the originals may be requested from the corresponding author.

### Image statistics

We computed FID with the pytorch-gan-metrics library. For generator comparisons, we used 50,000 images per condition: ImageNet validation photographs; DeePSim samples from an isotropic Gaussian; BigGAN class-conditioned samples (50 per class); and BigGAN with randomized class vectors. Additional datasets (COCO, THINGS) provided benchmarks. Low-level image statistics were computed in MATLAB for luminance, contrast, sharpness, color, symmetry, entropy, edge density and frequency power. Full formulas, functions and parameter values are listed in Supplementary Information.

### Closed-loop optimization (evolutions)

Each paired evolution session targeted a single visually responsive site (‘driver’). After receptive field mapping to place stimuli, two parallel threads were run in alternating image blocks: one in DeePSim space and one in BigGAN space. Each generator started from 30 seed images with small-norm latent codes. Ten static reference images were interleaved across blocks for stability checks. During a few pilot studies, single-thread evolutions were also used. Images were presented once each in rapid serial visual presentation (on for 100 ms; off for 150 ms) while the animals maintained fixation. The scalar fitness for each image was the driver’s mean firing rate from 50–200 ms after onset. Fitness values and latent codes were returned to the optimizer, which proposed a new set of latent codes for the next block. Sessions ran 10–60 blocks and stopped when responses plateaued. The same closed-loop protocol was used in silico with CNN units for parameter tuning and control analyses.

### Optimizers and parameterization

We used CMA-ES for both generators. To adapt to BigGAN geometry, we tuned the sampling step on CNN units, then fixed it for in vivo experiments. A robust initial σ for BigGAN was 0.06–0.12; for DeePSim, it was 3.0. For DeePSim, we also used a HessianCMA^28^ variant restricted to the top ~500 eigen-dimensions, which improves sample efficiency without changing outcomes relative to the full 4,096-dimensional space. All other CMA-ES parameters were identical across generators. This matched optimizer ensured that any differences reflect neural alignment rather than search hyperparameters. Full settings and ablations are provided in Supplementary Information.

### Quantifying evolution convergence speed

We averaged optimization trajectories across sessions after aligning their length. Trajectories were extrapolated to match the length of the longest session by padding each trajectory with the mean of its last two blocks, and responses within each session were normalized by the maximum block-mean activation of that driver across both threads. We then pooled sessions and fit a curve to activation versus block number with Gaussian-process regression. Convergence time was defined as the first block at which the smoothed trajectory reached 80% of its session-wise maximum. Alternative normalizations and thresholds gave the same qualitative pattern.

### Analysis of dynamic neuronal responses

We used two complementary analyses to relate temporal firing patterns to the image optimization process.

#### Temporal attribution of activation changes

For each site, we computed block-wise mean PSTHs over the 0–200 ms post-stimulus window. The block with the highest mean firing rate between 50–200 ms was defined as the maximum block:

$$\hat{B}=\arg \mathop{\max }\limits_{B}\mathop{\sum }\limits_{t=50}^{200}{\mathrm{PSTH}}_{B}(t)$$

where ${\mathrm{PSTH}}_{B}(t)$ represents the firing rate at time *t* for block *B*.

We then calculated a normalized difference PSTH between this block and the first block to quantify activation change:

$$\Delta \mathrm{PSTH}(t)=\frac{{\mathrm{PSTH}}_{\hat{B}}(t)-{\mathrm{PSTH}}_{{B}_{0}}(t)}{{\sum }_{{t}^{{\prime} }=0}^{200}({\mathrm{PSTH}}_{\hat{B}}({t}^{{\prime} })-{\mathrm{PSTH}}_{{B}_{0}}({t}^{{\prime} }))}$$

PSTHs were binned into non-overlapping time windows (5–50 ms) to reduce noise, and results were consistent across bin widths of 5, 10, 20, 25 and 50 ms. The resulting attribution vectors indicated how different temporal windows contributed to the overall activity increase during optimization.

At the population level, we pooled successful evolution threads (non-paired) for all driver units within each cortical area, including only those for which the maximum firing rate in two consecutive generations exceeded that of the first two generations (*P* < 0.01). For each area, we compared attribution vectors from DeePSim and BigGAN using two-sample *t*-tests. To confirm robustness, we repeated the analysis with paired *t*-tests restricted to sessions for which both evolution threads succeeded. Results were consistent across tests and bin widths, confirming temporal differences in activation dynamics between the two models.

#### Time-window-specific evolutionary trajectory

We also examined firing trajectories across contiguous sub-windows of the 0–200 ms period. For each generator $G$, block $b$ and time bin $t$, we normalized the response by the session’s maximum block-averaged firing rate across both threads:

$${\mathrm{PSTH}}_{G,b}(t)=\frac{{\mathrm{PSTH}}_{G,b}(t)}{\mathop{\max }\limits_{G,b,t\in (50,200)}{\mathrm{PSTH}}_{G,b}(t)}$$

where ${\mathrm{PSTH}}_{G,b}(t)$ is the firing rate for generator $G$, block $b$, at time $t$. Neurons were grouped by cortical area (V1, V4, PIT), and mean trajectories (±s.e.m.) were compared between DeePSim and BigGAN using paired *t*-tests at each block with false discovery rate correction. The analysis was repeated across bin widths and inclusion criteria (for example, ‘all successful’ vs ‘both successful’ sessions) to verify that results were stable across parameters.

### Similarity of PSTHs

To quantify the distance between paired evolution PSTHs, we used two measures: one integrated the absolute area between the difference of the two curves, and the other computed the difference between the average level of the PSTH curve, equivalently integrating the signed area between the PSTH curves.

$${d}_{\mathrm{psth}}=\mathop{\int }\limits_{t}|{\bar{r}}_{\mathrm{DeePSim}}\left(t\right)-{\bar{r}}_{\mathrm{BigGAN}}\left(t\right){|dt}$$

$${d}_{\mathrm{act}}=\mathop{\int }\limits_{t}\left({\bar{r}}_{\mathrm{DeePSim}}\left(t\right)-{\bar{r}}_{\mathrm{BigGAN}}\left(t\right)\right){dt}$$

For both measures, we first normalized the PSTHs; that is, divided them by the max block-average firing rate for that neuron.

### Tuning landscapes

#### Latent axes discovery (Hessian decomposition)

We examined local tuning geometry in BigGAN’s latent space using monkeys A–D. After completing the paired evolution experiments, we averaged the optimized latent vectors of the BigGAN evolution from the final generation ($\bar{z}$) and computed orthogonal axes ${v}_{i}$ within the class and noise subspaces by Hessian decomposition.

Given an image similarity metric $D:{\mathcal{J}}\times {\mathcal{J}}{\mathscr{\to }}{\mathbb{R}}$ (for example, LPIPS or pixel mean squared error), we computed a $d\times d$ real symmetric matrix ${H}_{z0}$ using second-order differentiation:

$${{H}_{{z}_{0}}=\frac{{\partial }^{2}D\left(G\left({z}_{0}\right),G\left({z}_{0}+\delta z\right)\right)}{\partial \delta z\,\partial \delta z}|}_{\delta z=0}$$

This matrix represents the local Riemannian metric of image space pulled back to the latent space, where ${v}^{\top }{Hv}$ quantifies the image change when moving along unit vector $v$.

BigGAN’s 256-dimensional latent space includes separate 128-dimensional class and noise subspaces. We restricted the $H$ matrix to these two subspaces, resulting in sub-matrices ${H}_{\mathrm{class}}$ and ${H}_{\mathrm{noise}}$, representing image changes induced by moving within these subspaces:

$$\begin{array}{cc}{H}_{\mathrm{class}}=H\left[0:128,0:128\right], & {H}_{\mathrm{noise}}=H[128:256,128:256]\end{array}$$

We performed eigen decomposition of these sub-matrices:

$$\begin{array}{cc}{V}_{\mathrm{class}},{\varLambda }_{\mathrm{class}}={\mathrm{eig}}\left({H}_{\mathrm{class}}\right), & {V}_{\mathrm{noise}},{\varLambda }_{\mathrm{noise}}={\mathrm{eig}}({H}_{\mathrm{noise}})\end{array}$$

The eigenvectors $\{{v}_{{\mathrm{class}},i}\},\{{v}_{{\mathrm{noise}},i}\}$ and eigenvalues $\{{\lambda }_{{\mathrm{class}},i}\},\{{\lambda }_{{\mathrm{noise}},i}\}$ provide principal directions and magnitudes of latent space traversal, whereby large eigenvalue directions maximize local image changes. Top eigenvectors in class and noise spaces generally correspond to interpretable image transformations^28^, while lower eigenvectors generally induce less semantically meaningful changes. Comparing the two subspaces, class traversal often changes object category, whereas noise traversal affects nuisance variables such as pose or size.

#### Line search algorithm

To sample latent vectors and images along axes ${v}_{i}$, we developed a one-dimensional line search algorithm. Owing to GAN latent space geometry, equal Euclidean or angular distances in latent space do not correspond to equal changes in image space. To ensure consistent image changes across axes, we applied a binary search algorithm to find latent traversal $\alpha$ achieving a target image change ${d}_{\mathrm{target}}$:

$$\alpha \left(+{d}_{\mathrm{target}}\right)={{\arg}}\mathop{\min }\limits_{\alpha > 0}\left|D\left(G\left(\bar{z}\right),G\left(\bar{z}+\alpha {v}_{i}\right)\right)-{d}_{\mathrm{target}}\right|$$

$$\alpha \left(-{d}_{\mathrm{target}}\right)={{\arg}}\mathop{\min}\limits_{\alpha < 0}\left|D\left(G\left(\bar{z}\right),G\left(\bar{z}+\alpha {v}_{i}\right)\right)-{d}_{\mathrm{target}}\right|.$$

This process generated image sequences passing through the center vector $$\bar{z}$$, with nine distances: $$[\mathrm{0.40,0.32,0.24,0.16,0},$$$$-0.16,-0.24,-0.36,-0.40]$$. Distances $$\pm 0.08$$ were excluded because of subtle image changes.

#### Stimuli selection and presentation

We selected ten eigenvectors from both class and noise spaces and used the line search algorithm to generate images corresponding to the nine distances along each axis. Images at the center location ($d=0$) were generated using $G(\bar{z})$, the mean latent vector from the last generation. Across 37 sessions with over 50 repetitions of the center image, only three channels showed a significant trend in firing rate as a function of repetition, all with slightly positive slopes. Thus, repetition suppression effects were minimal. Images were presented five to seven times per session, and mean firing rates were analyzed within a 50–200 ms post-image onset window.

#### Statistical analysis

Neuronal responses were recorded for each axis ${v}_{i}$ at signed distances ${d}_{k}$, resulting in a response dataset $\left\{{r}_{j}\left({d}_{k}\right)\right\}$. We performed one-way ANOVA to evaluate response modulation across distances. The peak location ${d}_{\max }$ was determined as:

$${d}_{\max }={{{\arg }}\max }_{d}\bar{r}(d)$$

where $\bar{r}(d)$ is the trial-averaged response.

Tuning curves were classified by their shapes using linear regression, one-dimensional Gaussian curve fitting and Gaussian-process regression. Unimodal curves with maxima not at $d=\pm 0.4$ were categorized as bell-shaped, while monotonic curves were classified as ramp-shaped. Fractions of significant bell-shaped and ramp-shaped curves were computed.

### Similarity of images and spatial attribution masks

#### Spatial attribution masks

To identify spatial regions in synthetic images most responsible for driving neural responses, we applied a spatial attribution analysis using a CNN (AlexNet). For each experimental session, we created an image dataset with all the evolution images sampled during the optimization process to drive specific neural sites and reference images interleaved throughout the experiment. These were processed through AlexNet to extract features from the last convolutional layer (*conv5*), which resulted in a four-dimensional feature tensor $F$ of shape $(N,C,H,W)$ (image number, channel number, height of feature map, width of feature map). We treated the feature vectors $F[:,:,i,j]$ at each spatial location $[i,j]$ as representations of the corresponding local image patches. Consequently, the predictive power of these features at each location served as a proxy for the spatial dependencies in neuronal activity. For each spatial location $[i,j]$ in the feature map, we fit a separate linear regression model (using fitlm.m), predicting the neural responses based on the extracted features across channels $F[:,:,i,j]$. The goodness-of-fit (adjusted ${R}^{2}$) for each spatial location was recorded, yielding a map of the model’s predictive power for each image. Higher ${R}^{2}$ values in specific regions of the feature maps indicated the locations most strongly associated with neural activity.

To quantify the visual feature similarity of spatial attribution maps across experiments for the same versus different drivers, we analyzed the linear weights from the spatial regression models. For each experimental thread, we used the weight maps ($W[:,:,i,j]$) derived from linear fits to features extracted from the last convolutional layer (conv5) of AlexNet. To focus on the most relevant weights, we thresholded the corresponding goodness-of-fit (adjusted ${R}^{2}$) maps at the 80th percentile and identified the largest connected region in the post-threshold map using regionprops.m. The linear weights within this region were averaged to generate a representative weight vector for each evolution. Then, we computed pairwise-Pearson correlations of weight vectors between threads within the same driving channel (‘same driver’) and between threads associated with randomly selected non-driving channels (‘random driver’). These pairwise correlations served as a measure of image similarity in the spatial attribution maps.

We also measured the smoothness of each spatial attribution map using total variation. Total variation quantifies the smoothness of a matrix by measuring the magnitude of gradients across its surface. For each spatial attribution map, total variation was computed as the sum of the Euclidean norms of the gradient vectors at each matrix point. Smaller total variations indicate a smoother map with fewer abrupt changes. Higher total variation values indicate a noisier map with more frequent localized variations.

Image similarity was also computed in different feature spaces, such as ResNet50 (robust), and using the LPIPS metric^37^. The LPIPS metric was instantiated with the AlexNet backbone.

#### Other analyses of local image similarity

LPIPS returns a spatial map of perceptual distances that is the same size as the image inputs. This metric was first tested using CNN-unit-driven evolutions; specifically, AlexNet convolutional layer 5 ($n=91$ randomly sampled units). For each paired evolution experiment (given one CNN unit), we sampled 15 images from the final generation of DeePSim images and 15 images from the final generation of BigGAN images, and then compared each of the DeePSim images to the other 15 BigGAN images (we also used BigGAN as references with the same results). The core of our analysis involved calculating the perceptual similarity between each image pair. After we obtained the LPIPS distance map, we converted it into a similarity heatmap by subtracting it from 1 (similarity = 1 – LPIPS). A key part of our analysis involved quantifying the concentration of perceptual similarity within the heatmaps. To do so, we calculated a concentration score from a given heatmap. The concentration score served as a quantitative measure of the degree to which perceptual similarity was localized within specific regions of the heatmap. The concentration index function defines a range of filter sizes to be applied to the heatmap, starting from a minimum size (set to 1 for individual pixel consideration), with multiple ranges, allowing the function to assess concentration at various spatial scales. For each filter size within the specified range, the function created a uniform filter (a matrix of ones) of that size. This filter was then normalized to ensure that its total sum was 1, maintaining the scale of the original heatmap values. The normalized filter was convolved with the heatmap, effectively averaging the heatmap values over areas corresponding to the filter size. This convolution process was repeated for each filter size in the range. After each convolution, the maximum value from the convolution result was extracted and stored. These maximum values represented the highest concentration of perceptual similarity for each filter size, indicating the presence of localized high-similarity regions within the heatmap. The concentration score was computed as the mean of these maximum values. This score provided a single, comprehensive metric indicating the extent of localized perceptual similarity in the heatmap. A higher concentration score suggested more pronounced localization of similarity, while a lower score indicated more diffuse similarity across the image. Next, for each reference DeePSim image for a given unit ${u}_{i}$, we randomly sampled another experiment and compared the unit ${u}_{i}$-generated DeePSim image to 15 of the random unit ${u}_{i}$-generated BigGAN images. We then compared the average similarity between the same driver image pairs (${u}_{i}$-generated DeePSim image vs ${u}_{i}$-generated BigGAN image) to the average similarity between the different-driver image pairs (${u}_{i}$-generated DeePSim image vs ${u}_{j}$-generated BigGAN image) and tested for statistical significance using a Wilcoxon signed-rank test.

### Feature attribution of evolved images

To further emphasize image features that were important during the evolution (evolution exemplars), we used a previously published method^38^. We used every pair of generated images and their associated neuronal response during a given evolution to build an encoding model. We found all the model units that correlated with the given neuronal firing rate. We factorized the correlation matrix and used it as an initialization for weights, which were then optimized as a factorized read-out weight matrix, following previous work^68^. We then used gradient-based optimization to find the maximum firing rate stimulus for the model unit; the optimized image was called a feature exemplar.

### Reporting summary

Further information on research design is available in the Nature Portfolio Reporting Summary linked to this article.

## Online content

Any methods, additional references, Nature Portfolio reporting summaries, source data, extended data, supplementary information, acknowledgements, peer review information; details of author contributions and competing interests; and statements of data and code availability are available at 10.1038/s41593-026-02207-1.

## Supplementary information

Supplementary Information Two formal treatments of the concept of ‘Alignment’ and ‘Manifold’, some extended Methods.

Reporting Summary

## Source data

Source Data Fig. 2 Response rate data in csv and pkl format to reproduce Fig. 2b. Original full-resolution images in png format for Fig. 2c. PSTH data in csv and pkl format for Fig. 2d.

Source Data Fig. 3 Similarity scores for Fig. 3e. Image similarity and PSTH similarity score for Fig. 3g.

Source Data Fig. 4 Table of success count of each thread for Fig. 4a. Max normalized optimization trajectory (mean and s.e.m.) of BigGAN and DeePSim thread to reproduce Fig. 4b and Fig. 4c. Evolution time constant table to reproduce Fig. 4d.

Source Data Fig. 5 Average PSTH for each session and thread before or after optimization Fig. 5a. Response rate for each 10 ms time window in csv format to reproduce Fig. 5c.

Source Data Fig. 6 Single trial neuronal responses for Monkey B channel 5b, for tuning curve fitting and plotting of Fig. 6c. Tuning curves along each Hessian eigen-dimensions for Monkey C channel 68B, and Monkey A channel 57, for Fig. 6d. All tuning curves (across all four monkeys) and their linear, Gaussian and GPR fitting statistics and classification as bell shape, ramp shape tuning for Fig. 6e.

Source Data Extended Data Fig. 1 Data for swarm plots.

Source Data Extended Data Fig. 2 Data for swarm plots.

Source Data Extended Data Fig. 4 Attribution data for time windows 5, 10, 20, 25 and 50 ms, for panel b. Time window specific evolution trajectory of time windows 10, 20, 25 and 50 ms, for panel c.

Source Data Extended Data Fig. 5 Data for the optimization experiments using Adam and CMA-ES on RealNVP transformed data. (Extended Fig. 5c). R2 data for linear predicting deep network hidden units with DeePSim and BigGAN latent code (Extended Fig. 5d,e).

## Figures and Tables

**Extended Data Table. 1:**
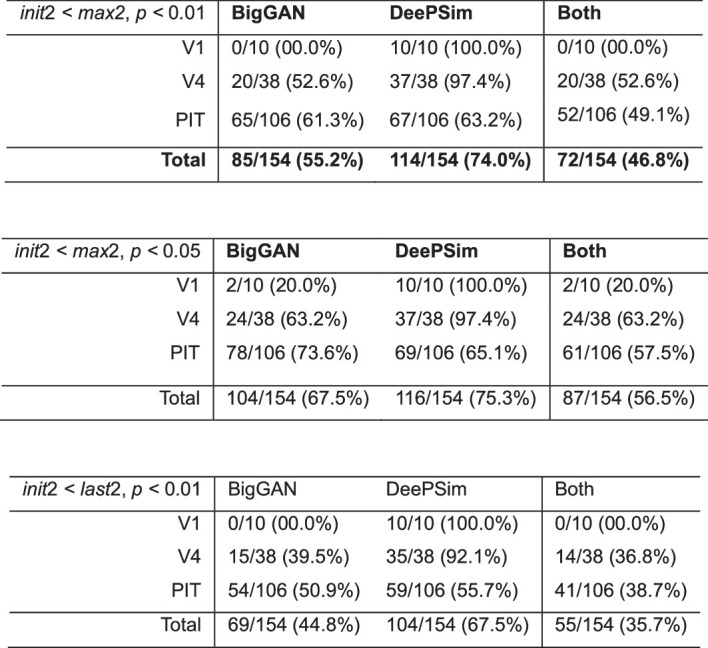
Success rates of evolution experiments per alternative criterion

Success criterion defined as an increase in activation from the first two generations (N = 25-40 images per generation) to two neighboring generations during the session, with statistical reliability estimated using a Student’s t-test. “Both” indicates that for a given session, both optimizers (BigGAN and DeePSim) were successful in their respective generative spaces. (Top): t-test between the activations in the first two blocks and maximally activating two blocks, init2 < max2, p < 0.01; (Middle): t-test between the activations in the first two blocks and maximally activating two blocks, init2 < max2, p < 0.05; (Bottom): t-test between the activations in the first two blocks and last two blocks init2 < last2, p < 0.01. All tests were two-sided.

**Extended Data Table. 2:**
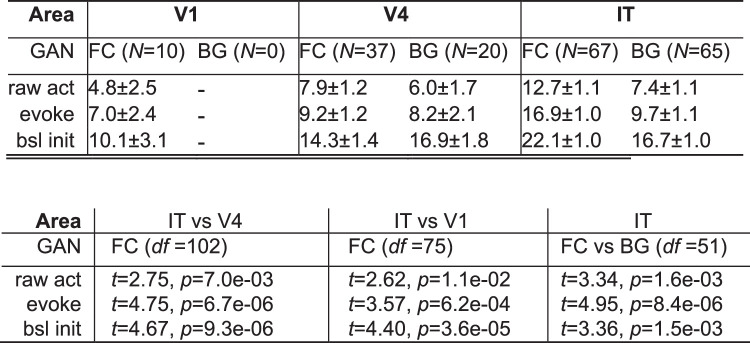
Convergence time statistics for each visual area and statistical tests

**Upper**, mean ± SEM of the statistics. Only threads with successful evolution in that space were included. (max > init, p < 0.01, two-sided) **Lower**: Significant test for the comparisons presented in the main paper with alternative statistics. Independent t-test for IT vs V4 and IT vs V1, paired t-test for FC vs BG in IT. **Abbreviations**: *raw act*: raw firing rate in 50-200 ms window; *evoke*: raw firing rate subtracting a session averaged baseline firing rate computed from 0-40 ms window; *bsl init*: raw firing rate subtracting the mean firing rate in the initial block, namely, activation increase from the initial block. We reported the bsl init in the main text and figure.

## Data Availability

Processed data for the analysis and reproduction of the figures can be downloaded at https://osf.io/pre96. The complete raw dataset used in this study is available upon request to C.R.P. Source data are provided with this paper.

## Code availability

The code used to analyze data in this paper is available on GitHub at https://github.com/Animadversio/Dynamic-Neuron-GAN-Alignment.
