# Supplementary material for: Neuronal tuning aligns dynamically with object and texture manifolds across the visual hierarchy
Source: Nat Neurosci. 2026 Mar 10;29(4):864–75. doi: 10.1038/s41593-026-02207-1 (PMC13061647; doi:10.1038/s41593-026-02207-1)
Supplement: Supplementary file 1 — Two formal treatments of the concept of ‘Alignment’ and ‘Manifold’, some extended Methods. [file 41593_2026_2207_MOESM1_ESM.pdf]

nature neuroscience

Supplementary information

https://doi.org/10.1038/s41593-026-02207-1

Neuronal tuning aligns dynamically with object and texture manifolds across the visual hierarchy

In the format provided by the authors and unedited

A more formal treatment of alignment between neuronal tuning and generative model

Let us consider a neuronal tuning function $f: \mathcal{I} \to \mathbb{R}$ mapping images to scalar responses and a generative model $G: \mathbb{R}^d \to \mathcal{I}$ mapping latent codes to images. The objective of the evolution experiment is to optimize the composition of these two functions $f \circ G$, i.e., the neuronal tuning function with respect to the latent codes. When the composition $f \circ G: \mathbb{R}^d \to \mathbb{R}$ is relatively simple or even linear, then optimizing such a function should be easy and better "aligned" by our previous definition. Specifically, let us consider a special case, where given the same space (e.g., image space), different parametrizations could lead to different degrees of optimization difficulty. Certain parametrizations will make the landscape appear substantially simpler, thus more amenable to optimization.

Toy model 1: Gaussian Tuning Curve, invertible transforms.

For example, let us postulate a simple isotropic Gaussian tuning function $f: \mathbb{R}^d \to \mathbb{R}$. Generally, this is a simple landscape to climb. However, if we parametrize the space $\mathbb{R}^d$ using a highly nonlinear transform, $g: \mathbb{R}^d \to \mathbb{R}^d$, (even if this is invertible, with inverse transform g^{-1}), then optimizing the composed function of $f \circ g$ will become very difficult; even though a unique global optimum still exists, a gradient- or evolutionary-algorithm-driven optimizer could get lost in the transformed landscape (Response Fig. 18A). As a low-dimensional toy example, in an R^d space, we can instantiate an invertible nonlinear transform of the space g with a deep, real-valued non-volume preserving neural network (realNVP) transformations and linear orthogonal transforms $g = O_n \circ q_n \circ \cdots O_1 \circ q_1$. In this case, the depth of the deep network n roughly represents the nonlinearity of the invertible transform and the complexity of the landscape. We defined a standard Gaussian tuning function in $\mathbb{R}^d$, $f(x) = \exp(-\|x - \mu\|^2)$, which has a unique global maximum μ. The composed function $f \circ g$ also had a unique global maximum $g^{-1}(\mu)$. We tested the CMA-ES algorithm and gradient ascent algorithm to optimize the composed function $f \circ g$, as a function of the complexity of g, and dimensionality d of the problem. We found that even in this invertible network, the deeper the set of transformational stages, the harder it is for an optimizer to reach the universal peak of the original function. We elaborate below.

Toy model 2: Gaussian Tuning Curve in feature space, invertible transforms.

Now let us consider a more complex and realistic scenario, where the neuronal tuning function $f(x)$ is not a simple function with respect to x itself (i.e., image pixels), but it is simple with respect to certain parametrization or representation of x. For example, the axis code of face cells with respect to parameter of faces12,13, or the bell shape tuning maps of V1, V4 and PIT neurons with respect to latent code of DeePSim14, or the linear prediction of neuronal responses with deep neural network features15. Thus, we could consider the tuning function $f(x) = N \circ \phi$, where N represents simple tuning curves such as linear or Gaussian, while ϕ is a highly nonlinear mapping (such as those mapping pixels to deep features in deep neural networks). Then consider the composition of generator and tuning function, $f \circ G = N \circ \phi \circ G$. If the generator G and nonlinear function ϕ are inverse to each other, this composition reduces to a simple tuning function to latent variables N. Even if ϕ were not an exact inverse to G, but only partially, this would reduce the complexity of the composed mapping, i.e., making it more linear, which could make the optimization easier.

More Realistic Scenario: Tuning curve of CNN units, Deep generative model.

We did a simulation to test this mechanistically, using a pretrained CNN (CaffeNet) as model of visual hierarchy. We used BigGAN as our G and the activations of hidden units in the CNN as f. We examined the linearity of the mapping $f \circ G$ by approximating it with a Ridge regression. Specifically, we sampled a set of 5000 latent codes z_i and collect the corresponding responses $r_i = f(G(z_i))$, then we performed a Ridge regression with cross-validated regularization selection to predict r_i from latent code z_i. We reported the test R^2 on the 20% test split as an indicator of the linearity

of the mapping $f \circ G$. Since any mapping was approximately linear locally, we made sure to sample the latent codes to cover the whole diversity of the latent space (1000 classes, Gaussian noise). We found that the linearity of the composed mapping generally increased progressively along the depth of in silico visual hierarchy (Response Fig. 18B-E). Layers with rectifying activation functions (ReLU) exhibited reduced linearity in the composed mapping (96 units were selected per layer Spearman correlation of linearity index and layer depth was 0.628 ± 0.007 for non-ReLU layers, 0.536 ± 0.013 for ReLU layers). We think the increasing linearity of hidden units or neural activity in latent codes of BigGAN could explain the increasing facility to optimize BigGAN latent code with higher level visual neurons. Further, when we restricted the latent codes to the object class part $z[:128]$ and repeated the regression procedure, the progression of linearity remained; however, when we restricted the latent code to noise part, the progression disappeared, with the population mean R^2 of each layer centered around 0 (Spearman correlation 0.077 ± 0.016 for non-ReLU layers, and 0.037 ± 0.029 for ReLU layers). This showed that the mapping from class subspace to the hidden units' activation is generally more linear, and the mapping from noise subspace to hidden units' activation is generally more nonlinear. This might explain why the class subspace is generally more "steerable" than the noise subspace. We did the similar experiment where *G* is DeePSim generator (fc6), examined the linearity as a function of depth of visual hierarchy, and found no clear evidence of such progression.

Biological Interpretation of Alignment

Given these observations, one intriguing possibility is that the DeePSim generator comprises a simpler parametrization of images, so combining DeePSim and the implicit tuning functions of V1, V4 ($f_{V1,V4} \circ G_{DeePSim}$) seems like a smoother landscape and thus easier to optimize. In contrast, with BigGAN as a nonlinear parameterization of images, its composition with the tuning functions of V1, V4 neurons ($f_{V1,V4} \circ G_{BigGAN}$) becomes less amenable to optimization. However, the composition of BigGAN and tuning function of IT neurons ($f_{IT} \circ G_{BigGAN}$) is simpler and more amenable to optimization. So, in this picture, the neural code of IT neurons f_{IT} served as a better "inverse" of the generator G_{BigGAN}, since their composition effectively reduced the complexity of the landscape (i.e., the effective depth of the nonlinear transform). This interpretation calls into the literature of inverse graphics and inverse generative model, i.e., vision as a process to invert the generative process of images (here, GAN).

Supplemental Treatment for Extended Figure 6. Empirical exploration of the term "manifold" in DeePSim and BigGAN latent spaces.

We explored whether the properties of the DeePSim and BigGAN latent spaces can be considered true or approximate manifolds using classic mathematical definitions. These generative models map latent space vectors to images in pixel space, forming high-dimensional structures that can be analyzed through manifold related mathematical properties such as smoothness, regularity, and non-intersection (injectivity). Here, we provide empirical evidence to characterize these properties.

Smoothness and Differentiability. Since the mapping operations G are implemented by neural networks, which are compositions of continuous functions (e.g., linear, bilinear, convolution, ReLU, tanh), they are continuous. Further, most operations in neural networks, except for ReLU, are smooth (infinitely differentiable) functions. The ReLU function, $\phi(x) = \Pi(x) \cdot x$, where

$$\Pi(x) = f(x) = \begin{cases} 0, & x < 0 \\ x, & x \geq 0 \end{cases}$$

is piecewise smooth. So, as their composition, the mappings G in BigGAN and DeePSim generators are not differentiable everywhere, yet differentiable almost everywhere. Namely, they are non-differentiable only when ReLU hidden units $\phi(x)$ have pre-activations $x = 0$ exactly — a zero-measure set in the latent space.

Regularity (Full-rankness of Jacobian). In this context, regularity is an empirical property we have managed to test using tools developed in our previous publication[28]. Is this a property held by the manifolds in question? The result is a yes-and-no. Consider the mapping $G: \mathbb{R}^d \to \mathbb{R}^{H\times W\times 3}, z \to \mathcal{I}$, where its Jacobian is a matrix of shape $(3HW, d)$:

$$J_{z0} = \frac{dG(z)}{dz}|_{z0}, \quad J_{z0} \in R^{3HW \times d}$$

This matrix is generally hard to compute, especially with backward-mode differentiation, due to the high dimensionality of the pixel space. Thus, it is usually expensive to check the rank of this Jacobian. However, we developed an alternative method to compute the Jacobian inner product via the double differential of the squared ℓ_2 image distance:

$$H_{z_0} = J_{z_0}^T J_{z_0} = \frac{\partial^2 \|G(z_0) - G(z_0 + \delta z)\|^2}{\partial \delta z \, \partial \delta z} |_{\delta z = 0}$$

The inner product of the Jacobian (or Hessian) H_{z0} has eigenvalues which are squares of the singular values of J_{z0}. Thus, H_{z0} has the same rank as J_{z0}, as long as J_{z0} is a tall matrix ($3HW > d$). This condition is satisfied in the case of DeePSim and BigGAN.

For our purposes, it is sufficient to check the rank of H_{z0} everywhere in the latent space. Practically, it is impossible to sample latent vectors z_0 exhaustively in a continuous latent space. Therefore, we sampled many random latent vectors z_0, computed the matrix H_{z0} at those points, and performed eigen decomposition of each H_{z0}:

$$U_{z0}, \Lambda_{z0} = \text{eig}(H_{z_0})$$

where Λ_{z0} contains the eigenvalues and U_{z0} contains the corresponding eigenvectors.

The spectrum Λ_{z0} holds the rank information of H_{z0} and J_{z0}: when it contains zero eigenvalues, the Hessian and Jacobian are rank deficient, thus violating the full-rank requirements of a conventional manifold.

Note that, since H_{z0} is the inner product of the Jacobian matrix J_{z0}, its eigenvalues are non-negative. Due to numerical imprecision in computing small eigenvalues, we regard negative eigenvalues and eigenvalues smaller than a certain threshold as effectively zero.

For BigGAN, we sampled 1000 latent vectors, one per class, and computed Λ_{z0} at each point. The median and the 5% and 95% percentiles of the spectrum across all points are shown in Panel A (Panels A, C, and E show the

spectrum of the Jacobian inner product (Hessian) of BigGAN and DeePSim, both for the full space and the reduced 500-dimensional top eigenspace). The center line represents the median eigenvalue across the 1000 random points, while the shaded area indicates the 5%-95% percentile range.

Empirical exploration of the term "manifold" in DeePSim and BigGAN latent spaces, continued.

We found that most points (895/1000) had a full-rank Jacobian, i.e., no zero eigenvalues. Among the points with a non-full-rank Jacobian, they had, on average, 12.6 ± 12.7 out of 256 eigenvalues that were zero. This suggests that the BigGAN generator has a full-rank Jacobian across most of the latent space, though some points in the latent space violate the regularity property.

However, when we repeated the analysis for DeePSim, 0/1000 Hessians were full rank. At each sampled point, there were 1707 ± 27 out of 4096 eigenvalues equal to zero. With a threshold of 10^{-7}, there were 3376 ± 16 eigenvalues effectively equal to zero. (Panel D, like Panel B, shows histograms of the number of zero eigenvalues at each latent vector across 1000 samples. Different colors represent different threshold values.) Clearly, the DeePSim generator has a degenerate Jacobian "everywhere," violating the regularity condition of the manifold. However, in our previous work[28], we found that the Hessian matrices in the DeePSim latent space were correlated across the latent space, which created a shared null space that could be projected out.

For example, if we consider the top eigenspace as the reduced latent space, let $U = [\mathbf{u}_1, \mathbf{u}_2, \dots, \mathbf{u}_{500}]$ be the matrix combining the top 500 eigenvectors of the average Hessian matrix. Then U becomes a basis in the reduced latent space, and the coordinate on this basis can be considered our latent code $\bar{z}$. The generative model can then be written as $G(U\bar{z})$, where $\bar{z} \in \mathbb{R}^{500}$.

When we limit the latent space to the top 500 eigenspace, we found that the new Jacobian was full rank at all 1000 sampled points (Panel E). We attempted to rescue the BigGAN generator using the same procedure, but after projecting out 128 of the 256 dimensions, there were still 12 out of 1000 points that had 5.3 ± 5.6 null dimensions. This suggests that the null space of the BigGAN generator is more heterogeneous across the latent space and harder to fix with a single projection. Thus, as we empirically demonstrated, the regularity property is not always satisfied. For BigGAN, it is satisfied at 90% of the sampled points but violated at the remaining points. For DeePSim, it was violated at all sample points. However, we can rescue this by considering only the subspace spanned by the top 500 eigenvectors. In that case, the mapping $\bar{z} \to G(U\bar{z})$ will be regular everywhere.

Non-intersection property (injectivity). Injectivity is defined as $G(z_1) = G(z_2) \Rightarrow z_1 = z_2$, meaning that different latent vectors map to different images. As mentioned, we have not explicitly tested this property. One reason is that we believe the generators are unlikely to satisfy this non-intersection property due to the degenerate Jacobian described earlier. For the full DeePSim model, the non-intersection property is immediately violated because its Jacobian is not full rank everywhere. Consider a vector $\boldsymbol{v}$ in the null space of the Hessian or Jacobian. Then:

$$G(z) = G(z + \alpha \boldsymbol{v}), \forall \alpha \in \mathbb{R}$$

i.e., all vectors along the line of v are mapped to the same image. A numerical example illustrating this is shown in Panel F, which depicts violations of the non-intersection (or injective) property in DeePSim and BigGAN. Specifically, traveling along a null vector in the latent space by one unit, results in a negligible pixel mean-squared error (MSE) and effectively no perceptual change in the generated image. Similarly, for points in BigGAN's latent space with non-zero null directions, traveling along these directions will also produce identical images. Thus, we conclude that neither DeePSim nor BigGAN generators are injective and do not satisfy the non-intersection property.

In summary, it is apparent that these empirical manifolds, which relate neuronal activity to image generators, are not true manifolds in the strictest mathematical sense. However, there are enough overlapping properties to justify their continued use in this manuscript and in the visual neuroscience literature.

Supplementary Methods.

Image Statistics

To measure the FID score, we used the function *calculate_frechet_distance* from the library pytorch-gan-metrics.

The set of images used to calculate these statistics were:

1. ImageNet 50000 images from the ImageNet validation set.
2. DeePSim 50000 images generated from DeePSim FC6 network, the latent codes were sampled from an isotropic Gaussian distribution with std. 4.
3. BigGAN 50000 images, 50 images per class for the 1000 classes. For each class, we used the pretrained object vector *c* for that class and 50 noise vectors *z* sampled from a 128d truncated Gaussian with truncation 0.7.
4. BigGAN-RND generated from BigGAN with 50000 latent vectors sampled from 256d spherical Gaussian with std 0.08.

Additionally, we quantified low-level image feature values across both generators. First, we optimized images for units in AlexNet conv5 units (with receptive fields placed at the center, N = 91 units); a preferred image for each unit was concurrently optimized using the DeePSim- and the BigGAN generators, as described above. Analyses are based on all images in the last evolved generation (N = 3640 total). Each image was analyzed as follows:

Luminance. Each image was converted from RGB to grayscale, then computed the global mean of the matrix elements.

Contrast. First, each image was converted from RGB to grayscale. Then, the standard deviation of the pixel intensity values in the grayscale image was computed to represent the root-mean-square (RMS) contrast. The standard deviation calculation was performed using the MATLAB function *std.m* on the pixel intensity values of the grayscale image.

Spatial Frequency. We calculated the total power of the spatial frequency content and the edge density for each image in a given set. Each image was first converted from RGB to grayscale using the *rgb2gray.m* function. The 2D Fast Fourier Transform (FFT) of the grayscale image was then computed using the *fft2.m* function, and the power spectrum was obtained by taking the squared magnitude of the shifted Fourier transformed image using *fftshift.m*. The total power of the power spectrum was calculated as the sum of all squared magnitudes. Edges were detected using the Canny method with the *edge.m* function, and the edge density was calculated as the proportion of edge pixels to the total number of pixels in the image.

Texture. Texture features were computed for each image in a given set using two methods: Gray Level Co-occurrence Matrix (GLCM) and Local Binary Patterns (LBP). Each image was first converted to grayscale using the *rgb2gray.m* function. The GLCM was computed using the *graycomatrix.m* function, and its properties—contrast, correlation, energy, and homogeneity—were extracted using the *graycoprops.m* function. Additionally, LBP features were extracted using the *extractLBPFeatures.m* function, providing a descriptor of the local spatial structure and contrast of the image.

Color. Color distribution was computed for each image by measuring the histogram and balance for each color channel (red, green, and blue). For each image, the histogram of each color channel was computed using the *imhist.m* function. Additionally, the color balance for each channel was determined by calculating the mean intensity value.

Sharpness. We calculated the sharpness of each image in a given set by first converting the image to grayscale using the *rgb2gray.m* function if it was in color. We then used the Sobel operator to compute the gradient in both the X and Y directions, using convolution with the Sobel kernels defined in the *conv2.m* function. The gradient magnitudes

were obtained by computing the square root of the sum of the squares of the gradients in the X and Y directions. Finally, the sharpness was quantified as the mean magnitude of these gradients, providing a measure of the image's detail.

Shape Statistics. Other shape statistics were computed for each image by first converting the RGB images to grayscale. A binary image was then obtained through thresholding with the *graythresh.m* and *imbinarize.m* functions. The Euler number, representing the number of objects minus the number of holes, was calculated using the *bweuler.m* function. For each object in the binary image, we computed the aspect ratios of their bounding boxes (the ratio of width to height) using the *regionprops.m* function, focusing on the largest object detected by area.

Frequency Power Distribution. We calculated the frequency power distribution in vertical, horizontal, and diagonal directions for each image. Each image was first converted to grayscale, and we then applied the Fourier Transform and shifted the zero-frequency component to the center of the spectrum. The magnitude spectrum was obtained by taking the absolute value of the shifted Fourier Transform. We calculated the overall power as the sum of all magnitudes. The vertical power was computed as the sum of magnitudes along the vertical central line, the horizontal power as the sum of magnitudes along the horizontal central line, and the diagonal power as the sum of magnitudes along the diagonal line from the top-left to the bottom-right, each normalized by the overall power.

Symmetry. The symmetry of each image was calculated across the horizontal, vertical, and radial axes.

Each image was first converted to grayscale. Horizontal symmetry was assessed by comparing the top half of the image to the flipped bottom half, calculating the mean absolute difference between corresponding pixels. Vertical symmetry was evaluated similarly by comparing the left half to the flipped right half. Radial symmetry was determined by creating concentric rings of pixels around the center of the image and comparing each pixel in the ring to the mean pixel value of that ring, with the radial symmetry score being the average absolute difference from the mean.

Entropy. The Shannon entropy and joint entropy for each image were also computed. The Shannon entropy, which measures the information content of the grayscale representation, was computed by first converting each RGB image to grayscale and then applying the *entropy.m* function. The joint entropy, which measures the combined information content of the RGB channels, was computed by flattening the RGB channels and creating a joint histogram using the *imhist.m* function. The joint entropy was then calculated from this histogram by normalizing the counts and summing the product of the non-zero histogram values with their log base 2 values.

Optimizers and parameterization

We set out to make sure that the closed-loop neural-guided image synthesis system would work with newer generative networks such as BigGAN. This required establishing the appropriate *optimizer* to search in the latent space of the generator, as we did in previous work[1], where we found that Covariance Matrix Adaptation Evolutionary Strategy (CMA-ES) was particularly well-suited for neuron-driven image synthesis. CMA-ES is a zeroth-order optimization algorithm[2], so it does not require gradient information to guide its search. Instead, it relies solely on function evaluations—measuring the performance or fitness of candidate solutions. Still, developing closed-loop optimizers for visual cortex neurons is costly, so we first simulated the process with a more tractable synthetic problem, a common strategy in the field of evolutionary computing[3]. As image-computable models, convolutional neural networks (CNNs) serve as good first approximations of the primate visual system. We have shown that the tuning landscapes of CNN hidden units share many similar geometric properties as those of visual neurons. Thus, we first tuned the configurations of various evolutionary algorithms for BigGAN via CNN hidden units. The key parameter was the standard deviation of the sampling distribution (*i.e.,* exploration step size). This parameter controls the distance of exploration in the latent space, which translates to the distances between image samples. If the images within a batch sample were too similar to each other, the difference in neuronal response to images would be overwhelmed by single-trial response variability,

obscuring the "gradient" direction of the tuning landscape. On the other extreme, if the images within a batch were too far apart from each other, traveling in the average direction of their latent codes would not help. Optimally, the search step parameter should be tuned to suit the average *slope* of the neuron's tuning function. Heuristically, we found that a robust step size for BigGAN was 0.06-0.4, in contrast to 3.0 for CMA-ES. We tested these optimizers on *in silico* units from convolutional neural networks and confirmed that they could successfully optimize activation for these units. We then tested these parameters *in vivo*. Note that no other changes were done to the optimizer across image spaces.

For the DeePSim evolutions, we used the *Cholesky* covariance matrix adaptation evolutionary strategy (CMA-ES) algorithm and the *HessianCMA* algorithm. The Cholesky CMAES has the same parameters as in previous works: standard deviation was initialized as 3.0. For the *HessianCMA* Optimizer, we pre-computed the 500 most informative dimensions in the latent space of the DeePSim GAN through Hessian decomposition, and only optimized in that linear subspace, which was more sample efficient, and we have shown it yielded comparable activation with the optimizer operating in the full 4096D space. For the BigGAN evolutions, we used the Cholesky CMAES and *HessianCMA* algorithms with the optimized parameters optimized on *in silico* experiments, primarily choosing an initial standard deviation of 0.06, with up to 0.12 for *in silico* studies. To ensure consistency in our comparison, all experiments used the CMA-ES optimization algorithm for both BigGAN and DeePSim experiments, with adjustments to the initial step size to account for differences in the geometry of BigGAN's latent space. This adaptation prevented sampling from untrained regions of the latent space, while maintaining all other parameters consistent across experiments. By first optimizing these parameters in silico and freezing them for in vivo testing, we controlled for confounding factors, enabling a fair comparison of generative models across brain regions.

Methods-pertinent references

1. Wang, B. & Ponce, C. R. High-performance Evolutionary Algorithms for Online Neuron Control. *Genet. Evol. Comput. Conf.* https://doi.org/10.1145/3512290.3528725 (2022) doi:10.1145/3512290.3528725.
2. Hansen, N. & Ostermeier, A. Completely derandomized self-adaptation in evolution strategies. *Evol. Comput.* **9**, 159–195 (2001).
3. Long, F. X. *et al.* Learning the characteristics of engineering optimization problems with applications in automotive crash. in *Proceedings of the Genetic and Evolutionary Computation Conference* 1227–1236 (Association for Computing Machinery, New York, NY, USA, 2022). doi:10.1145/3512290.3528712.
