# Supplementary material for: Neuronal tuning aligns dynamically with object and texture manifolds across the visual hierarchy
Source: Nat Neurosci. 2026 Mar 10;29(4):864–75. doi: 10.1038/s41593-026-02207-1 (PMC13061647; doi:10.1038/s41593-026-02207-1)
Supplement: Supplementary file 2 — Reporting Summary [file 41593_2026_2207_MOESM2_ESM.pdf]

nature portfolio

Corresponding author(s): Carlos R. Ponce

Last updated by author(s): Nov 1, 2025

# Reporting Summary

Nature Portfolio wishes to improve the reproducibility of the work that we publish. This form provides structure for consistency and transparency in reporting. For further information on Nature Portfolio policies, see our Editorial Policies and the Editorial Policy Checklist.

## Statistics

For all statistical analyses, confirm that the following items are present in the figure legend, table legend, main text, or Methods section.

| n/a | Confirmed | |
|---|---|---|
| ☐ | ☒ | The exact sample size (*n*) for each experimental group/condition, given as a discrete number and unit of measurement |
| ☐ | ☒ | A statement on whether measurements were taken from distinct samples or whether the same sample was measured repeatedly |
| ☐ | ☒ | The statistical test(s) used AND whether they are one- or two-sided<br>*Only common tests should be described solely by name; describe more complex techniques in the Methods section.* |
| ☒ | ☐ | A description of all covariates tested |
| ☒ | ☐ | A description of any assumptions or corrections, such as tests of normality and adjustment for multiple comparisons |
| ☐ | ☒ | A full description of the statistical parameters including central tendency (e.g. means) or other basic estimates (e.g. regression coefficient) AND variation (e.g. standard deviation) or associated estimates of uncertainty (e.g. confidence intervals) |
| ☐ | ☒ | For null hypothesis testing, the test statistic (e.g. *F*, *t*, *r*) with confidence intervals, effect sizes, degrees of freedom and *P* value noted<br>*Give P values as exact values whenever suitable.* |
| ☒ | ☐ | For Bayesian analysis, information on the choice of priors and Markov chain Monte Carlo settings |
| ☒ | ☐ | For hierarchical and complex designs, identification of the appropriate level for tests and full reporting of outcomes |
| ☐ | ☒ | Estimates of effect sizes (e.g. Cohen's *d*, Pearson's *r*), indicating how they were calculated |

*Our web collection on statistics for biologists contains articles on many of the points above.*

## Software and code

Policy information about availability of computer code

| | |
|---|---|
| Data collection | Experimental control and stimulus generation were performed using MATLAB (R2020b–R2024a) together with MonkeyLogic 2.0and NIMHML toolboxes, as well as Python (3.7–3.10) with PyTorch (1.7–2.0) and the pytorch-pretrained-biggan package (v0.1.0). Closed-loop optimization used Hansen’s CMA-ES implementation (2016–2018 release) with in-lab modifications. Neural data were acquired using the Plexon OmniPlex recording system and PlexControl software (v1.16–1.18). Image analysis and model-fitting relied on NumPy (1.19–1.26), SciPy (1.5–1.11), torchvision (0.8–0.15), lpips (0.1), and OpenCV (4.5–4.9). Eye position was monitored using an ISCAN infrared eye-tracking system. |
| Data analysis | Matlab<br>Python<br>Code available at<br>https://github.com/Animadversio/Dynamic-Neuron-GAN-Alignment |

For manuscripts utilizing custom algorithms or software that are central to the research but not yet described in published literature, software must be made available to editors and reviewers. We strongly encourage code deposition in a community repository (e.g. GitHub). See the Nature Portfolio guidelines for submitting code & software for further information.

## Data

Policy information about availability of data

All manuscripts must include a data availability statement. This statement should provide the following information, where applicable:

- Accession codes, unique identifiers, or web links for publicly available datasets
- A description of any restrictions on data availability
- For clinical datasets or third party data, please ensure that the statement adheres to our policy

At this time, we are not releasing the complete raw dataset because we are still conducting ongoing analyses that depend directly on the full set of neuronal recordings. These involve characterizing population activity during the closed-loop evolution process and examining long-term dynamics and stability across days and sessions. Since this study requires access to the unprocessed continuous recordings and full metadata, we cannot publicly post the raw files without compromising the integrity of the ongoing work. Processed data sufficient to reproduce all figures in the present manuscript have been deposited in OSF (https://osf.io/pre96). The complete raw dataset will be made available once these population-level analyses are finished. In the meantime, the raw data can be provided upon reasonable request to the authors.

## Research involving human participants, their data, or biological material

Policy information about studies with human participants or human data. See also policy information about sex, gender (identity/presentation), and sexual orientation and race, ethnicity and racism.

| | |
|---|---|
| Reporting on sex and gender | NA |
| Reporting on race, ethnicity, or other socially relevant groupings | NA |
| Population characteristics | NA |
| Recruitment | NA |
| Ethics oversight | NA |

Note that full information on the approval of the study protocol must also be provided in the manuscript.

# Field-specific reporting

Please select the one below that is the best fit for your research. If you are not sure, read the appropriate sections before making your selection.

☒ Life sciences ☐ Behavioural & social sciences ☐ Ecological, evolutionary & environmental sciences

For a reference copy of the document with all sections, see nature.com/documents/nr-reporting-summary-flat.pdf

# Life sciences study design

All studies must disclose on these points even when the disclosure is negative.

| | |
|---|---|
| Sample size | We worked with four primates and conducted more than 200 recording sessions. The primary unit of analysis was an individual neuron or neuronal microcluster. We did not use a formal a priori sample size calculation. Instead, sample size expectations were guided by computational simulations from our previous work, which indicated that the optimization algorithms would produce measurable changes in firing rate given the planned number of generations, search parameters, and expected neuronal variability. Data collected in the present study confirmed that the observed effects were robust at this scale, supporting that the sample sizes were sufficient for the analyses performed. Experiments were replicated across independent recording sessions and neuronal sites. Monkeys A and B contributed 87 unique sites, and monkeys C and D contributed 160 additional unique sites, but only a subset was used for follow-up experiments. Each session–site combination was treated as an independent biological replicate. Multiple trials within a block and repeated blocks within an evolution were technical replicates and were averaged or used for trajectory fitting and not counted as independent samples. |
| Data exclusions | 16 experimental sessions were excluded due to unstable recording quality (e.g., when baseline firing rates changed in a manner consistent with signal degradation, as measured via a set of fixed reference images). Experiments were also excluded if they had fewer than 15 blocks. |
| Replication | We observed that the image optimization algorithm could be replicated across multiple cortical sites and visual areas within and across monkeys. Further, many results would be replicated in silico, and analyses where the computational models failed to replicate observed neurophysiological results, were included as key findings. |
| Randomization | There is no treatment group allocation in our study, as each subject was treated equally. No randomization was required for analysis. We used four animals, and they were allocated to the same group. By using chronically implanted arrays without prior functional imaging guidance, we had a random sampling of neuronal responses in cortex. All sites were tested using the same experimental protocol. This experimental protocol design used pseudo-random block presentation of images for image selectivity. The image synthesis experiments were also based on stochastic algorithms, and depended on each neuronal site responses. The use of four animals allowed for covariate control, |

| | |
|---|---|
| | because the inability to replicate results in one animal's neuronal population using a separate animal's neuronal population would suggest that the effects are specific to the particular animal. |
| Blinding | There is no treatment group allocation in our study. No blinding was required for analysis. |

# Reporting for specific materials, systems and methods

We require information from authors about some types of materials, experimental systems and methods used in many studies. Here, indicate whether each material, system or method listed is relevant to your study. If you are not sure if a list item applies to your research, read the appropriate section before selecting a response.

## Materials & experimental systems

| n/a | Involved in the study |
|---|---|
| ☒ | ☐ Antibodies |
| ☒ | ☐ Eukaryotic cell lines |
| ☒ | ☐ Palaeontology and archaeology |
| ☐ | ☒ Animals and other organisms |
| ☒ | ☐ Clinical data |
| ☒ | ☐ Dual use research of concern |
| ☒ | ☐ Plants |

## Methods

| n/a | Involved in the study |
|---|---|
| ☒ | ☐ ChIP-seq |
| ☒ | ☐ Flow cytometry |
| ☒ | ☐ MRI-based neuroimaging |

## Animals and other research organisms

Policy information about studies involving animals; ARRIVE guidelines recommended for reporting animal research, and Sex and Gender in Research

| | |
|---|---|
| Laboratory animals | Four male adult macaques (8- to 12-years-old were in the study |
| Wild animals | Lab-born |
| Reporting on sex | The study was only conducted on male macaques, which were our only available sex |
| Field-collected samples | NA |
| Ethics oversight | All procedures were approved by the Institutional Animal Care and Use Committees at Harvard Medical School and Washington University School of Medicine. |

Note that full information on the approval of the study protocol must also be provided in the manuscript.

## Plants

| | |
|---|---|
| Seed stocks | NA |
| Novel plant genotypes | NA |
| Authentication | NA |
