# Supplementary figures and images for: Neuronal tuning aligns dynamically with object and texture manifolds across the visual hierarchy
Source: Nat Neurosci. 2026 Mar 10;29(4):864–75. doi: 10.1038/s41593-026-02207-1 (PMC13061647; doi:10.1038/s41593-026-02207-1)

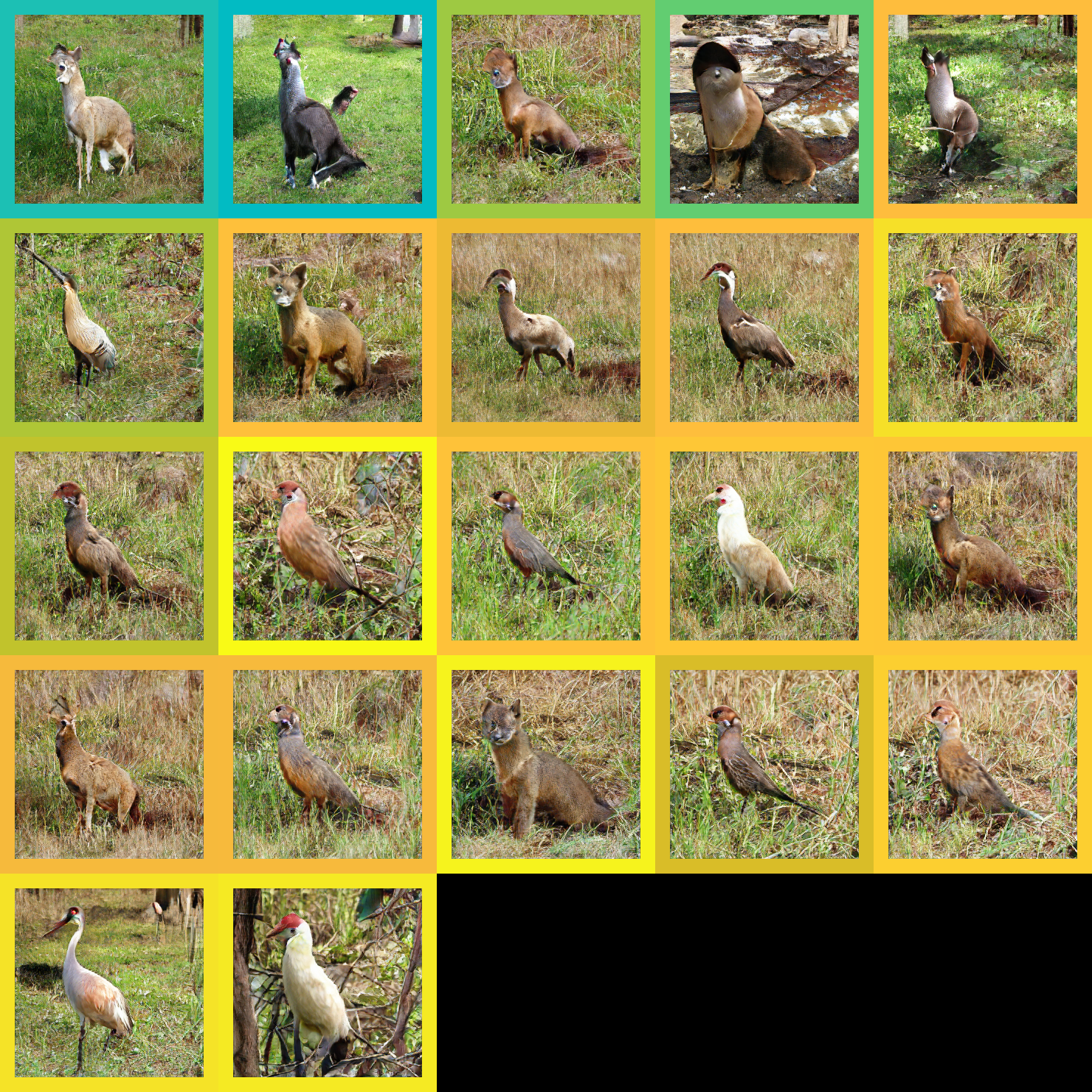

Supplement: Supplementary file 3 — Response rate data in csv and pkl format to reproduce Fig. 2b. Original full-resolution images in png format for Fig. 2c. PSTH data in csv and pkl format for Fig. 2d. [file 41593_2026_2207_MOESM3_ESM.zip › Both_Exp155_BGImageEvol_best_framed.png]

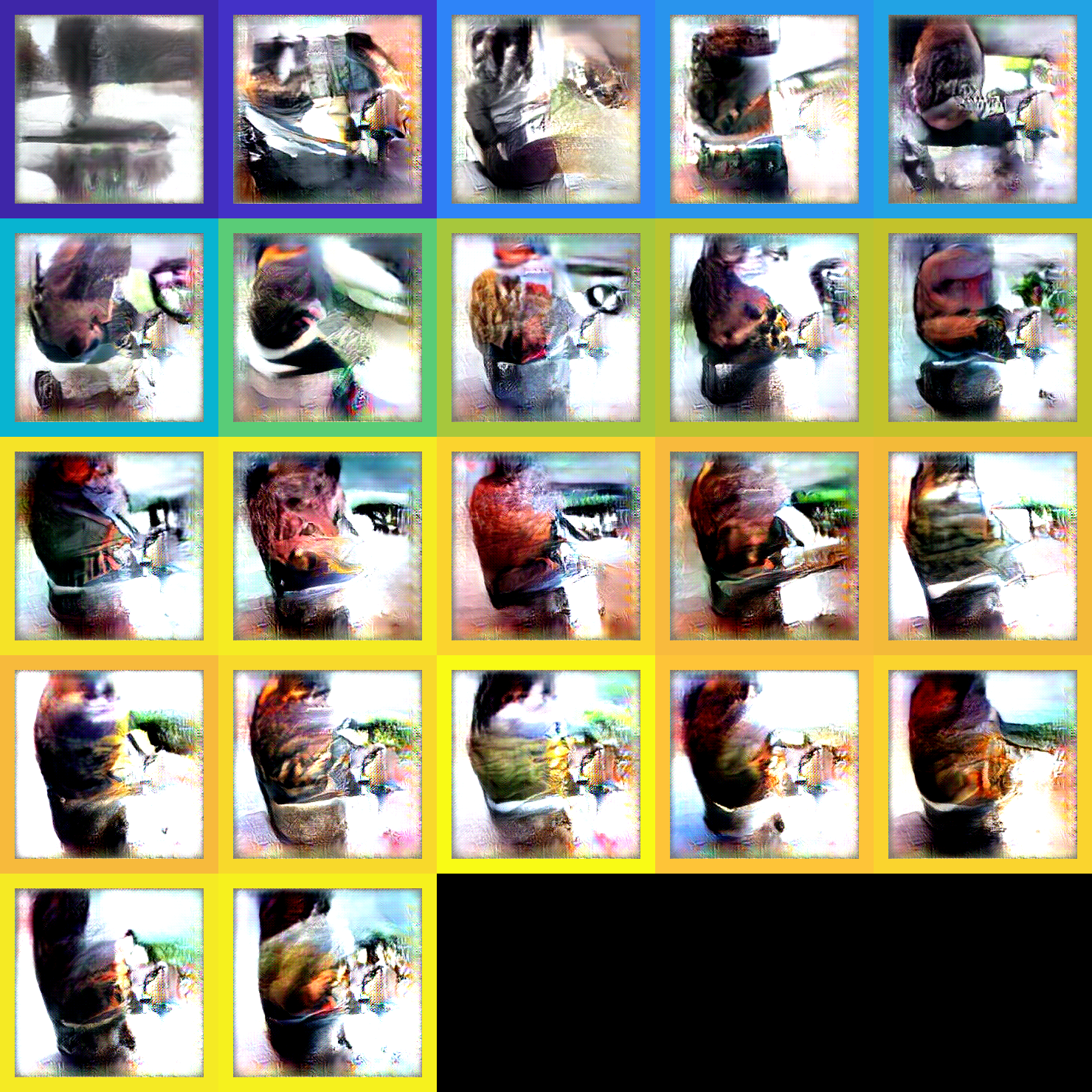

Supplement: Supplementary file 3 — Response rate data in csv and pkl format to reproduce Fig. 2b. Original full-resolution images in png format for Fig. 2c. PSTH data in csv and pkl format for Fig. 2d. [file 41593_2026_2207_MOESM3_ESM.zip › Both_Exp155_FC6ImageEvol_best_framed.png]
